# Pharmacodynamic-Driven Sequence-Dependent Synergy Effects in Pemetrexed-Osimertinib Combination Against Non-Small Cell Lung Cancer (NSCLC): Optimizing Synergy Through Sequential Interval

**DOI:** 10.3390/pharmaceutics17081044

**Published:** 2025-08-12

**Authors:** Kuan Hu, Yu Xia, Tong Yuan, Yan Lin, Jin Yang

**Affiliations:** Center of Drug Metabolism and Pharmacokinetics, China Pharmaceutical University, Nanjing 211198, China; 3119010074@stu.cpu.edu.cn (K.H.); 3320011002@stu.cpu.edu.cn (Y.X.); 3121010078@stu.cpu.edu.cn (T.Y.)

**Keywords:** pemetrexed, Osimertinib, synergy effect, sequential drug administration, schedule-dependent

## Abstract

**Background:** Combining pemetrexed (PEM) with Osimertinib (OSI) improves outcomes in epidermal growth factor receptor (EGFR)-mutant non-small cell lung cancer (NSCLC), but optimal scheduling remains undefined. Sequential PEM → OSI strategies may outperform concurrent administration; however, the critical dosing interval determining synergy has not been explored. **Methods**: PEM pharmacodynamics were divided into an OSI-antagonized early phase (S-phase arrest and DNA damage accumulation) and OSI-synergized late phase (DNA damage peak, apoptosis initiation, and feedback EGFR activation). Time-course profiling of cell cycle, DNA damage, apoptosis, and EGFR pathways was evaluated under monotherapy or sequential combination regimens to elucidate the mechanisms underlying synergistic/antagonistic effects. **Results:** OSI antagonizes PEM’s early phase via G1 arrest but potently enhances late-phase apoptosis through Rad51/thymidylate synthase suppression, Bim upregulation, and inhibition of EGFR signaling. The 48 h interval PEM → OSI uniquely enabled complete early-phase execution and aligned OSI exposure with late-phase initiation, yielding robust synergy across OSI-sensitive cell lines. In contrast, the 24 h interval PEM → OSI sequence demonstrated synergy only in PEM-sensitive PC9 cells. Both concurrent PEM + OSI and OSI → PEM sequence induced attenuated DNA damage and apoptotic signaling. **Conclusions:** The 48 h interval PEM → OSI sequence maximizes efficacy by temporally segregating antagonistic and synergistic interactions. This pharmacodynamically optimized regimen represents a promising strategy for clinical translation.

## 1. Introduction

Primary lung cancer exhibits the highest global incidence and mortality among all malignancies, with non-small cell lung cancer (NSCLC) constituting 80% of cases [1]. EGFR-mutations are present in more than 50% of Asian and 10–20% of European patients with NSCLC [2,3], positioning EGFR tyrosine kinase inhibitors (EGFR-TKIs) as a cornerstone of NSCLC treatment, particularly in East Asian populations [2,3]. Prolonged EGFR-TKI therapy, such as gefitinib, induces resistance mutations, most notably T790M, which mediates approximately 50% of acquired resistance [4,5]. The third-generation EGFR-TKI Osimertinib (OSI), designed to target T790M resistance, demonstrates first-line efficacy, but ultimately fails to prevent disease progression in most patients [6]. This necessitates EGFR-TKI-based combination therapies to enhance the efficacy in EGFR-mutant populations [7]. Building on combinatorial precedents of gefitinib/erlotinib with chemotherapy (pemetrexed/platinum) [8,9], antiangiogenics (bevacizumab) [10,11,12,13], or immunotherapies (PD-1 inhibitors), OSI-based combination regimens are now under investigation. However, OSI combinations with bevacizumab showed no PFS benefit [14] and, with immunotherapy, raised safety concerns (interstitial pneumonia) [15].

In contrast, gefitinib combined with pemetrexed (PEM)-based chemotherapy improved both PFS and overall survival (OS) in NEJ009 and JMIT trials [8,9]. This synergy may stem from chemotherapy’s nontargeted mechanisms counteracting tumor heterogeneity to delay EGFR-TKI resistance [7,16]. For example, gefitinib–PEM combinations suppress T790M-mediated resistance and inhibit epithelial–mesenchymal transition (EMT) [4]. Given OSI’s superior efficacy over gefitinib [17], the combination of OSI with chemotherapy is hypothesized to yield greater clinical benefits [6]. In the ongoing FLAURA2 trial [6], OSI is combined with platinum-based chemotherapy for four induction cycles, then followed by maintenance PEM (every 3 weeks) and OSI (daily) combination. Interim results confirm significant PFS improvement, with immature OS data showing favorable trends [6]. Optimizing the combination strategy of OSI with platinum-based doublet therapy during the induction phase and with PEM during the maintenance phase represents a critical next-step direction. Sequential dosing regimens are posited to enhance efficacy over concurrent strategies, as evidenced clinically [18,19,20]. For instance, sequential administration of PEM with almonertinib (a third-generation EGFR-TKI) outperformed concurrent administration [18]. A key hypothesis proposes that sequential PEM followed by EGFR-TKI separates the timing of two critical events: PEM-induced S-phase arrest (essential for PEM’s cytotoxicity) and EGFR-TKI-induced G1 arrest. This temporal separation prevents interference because G1 arrest may protect tumor cells from PEM’s cytotoxicity effects [21], thereby allowing uncompromised PEM efficacy. However, the optimal duration of PEM treatment prior to EGFR-TKI exposure (i.e., sequential interval) remains unexplored [21]. An interval that is too short may result in suboptimal PEM efficacy due to premature G1 arrest-mediated antagonism, ultimately diminishing synergy. Conversely, an excessively long interval not only prevents EGFR-TKI from coinciding with the peak effect of PEM (thereby compromising synergy) but also reduces effective OSI exposure time. In the 28 d PEM → aumolertinib regimen (day 1 PEM; days 8–28 aumolertinib) [18], such a long sequential interval reduced aumolertinib exposure by 25% compared to concurrent dosing.

In addition to G1 arrest-mediated antagonism, EGFR-TKI also exhibits synergistic effects on PEM, which can be summarized in three aspects based on previous research. First, PEM induces feedback activation of EGFR and its downstream subpathways, and this feedback activation is subsequently inhibited by EGFR-TKIs [19,21,22]. However, which downstream subpathway (ERK1/2 [19] or Akt [21,22]) exhibits more pronounced compensatory upregulation remains debated. Moreover, no studies have experimentally confirmed if suppressing this feedback fully explains EGFR-TKI-mediated synergy with PEM. Second, EGFR-TKIs are reported to downregulate thymidylate synthase (TS) [4,22], a known sensitivity marker for PEM [23,24]. Despite this association, whether TS down-regulation enhances PEM efficacy even after PEM was removed in the PEM → EGFR-TKI sequence remains unverified [4,22]. Third, in PEM → Icotinib (a first-generation EGFR-TKI) combinations, Icotinib enhances PEM-induced pro-apoptotic effects. Similar potentiation has likewise been observed with other EGFR-TKIs [4,16,19], although the mechanism by which EGFR-TKIs enhance PEM-induced pro-apoptotic effects remains unclear. Overall, the current study has the following limitations: (1) Researchers hypothesized mechanisms based on observed phenomena but did not systematically design experiments to validate them. (2) Failed to adequately address the contradiction between synergistic versus antagonistic effects of EGFR-TKI on PEM. (3) Failed to establish a relationship between the synergistic effects of EGFR-TKI on PEM and the administration sequence. (4) The direct mechanism by which EGFR-TKIs enhance PEM-induced pro-apoptotic effects remains unelucidated. (5) Critically, no existing studies have investigated the impact of sequential dosing intervals on the synergy effect.

In this study, to optimize the PEM → OSI combination strategy, we propose a hypothesis that divides PEM’s pharmacodynamics into an early phase (one-carbon/nucleotide depletion, S-phase arrest, replication fork stalling, and DNA damage accumulation) and a late phase (DNA damage peak, DNA damage-to-apoptosis transition, and feedback EGFR activation), where early-phase effect accumulation governs late-phase response intensity. OSI antagonizes PEM’s early phase through G1 arrest but enhances late-phase effects via Rad51/TS suppression, pro-apoptotic Bim upregulation, and EGFR signaling blockade. Once PEM progresses to the late phase, G1 arrest no longer antagonizes its effects. In both the OSI → PEM and PEM + OSI sequences, G1 arrest impairs early-phase efficacy and attenuates late-phase responses, resulting in antagonism. With a 24 h interval PEM → OSI (means PEM was treated for 24 h and then replaced with OSI), premature OSI interference truncates early-phase effects, such as incomplete S-phase arrest and DNA damage accumulation, yielding minimal synergy. Conversely, a 48 h interval ensures complete early-phase execution, achieving maximal DNA damage, and aligns OSI administration with the onset of late-phase events, including the DNA damage peak and apoptosis signaling initiation. This enables synergy via OSI-mediated enhancement of DNA damage-repair inhibition, pro-apoptotic effects, and suppression of feedback EGFR signaling while avoiding early-phase disruption. This hypothesis provides the clearest elucidation to date of PEM → EGFR-TKI synergy mechanisms [18,19,21,22] and establishes the 48 h interval PEM → OSI sequence as the pharmacodynamically optimized regimen.

## 2. Materials and Methods

### 2.1. Drug and Reagents

All drugs, reagents, and antibodies used in this study were commercially available; their catalog numbers and manufacturers are listed in Tables S1 and S2.

### 2.2. Cell Lines

Four EGFR-mutated human NSCLC cell lines—HCC827, PC-9, NCI-H1975, and NCI-H1650—were obtained from the Cell Bank of the Chinese Academy of Sciences (Shanghai, China). All cell lines were cultured in RPMI1640 (KeyGen BioTech, Nanjing, China) supplemented with 10% non-heat-inactivated fetal bovine serum (Procell, Wuhan, China), 1% GlutaMax, penicillin (100 U/mL), and streptomycin (100 U/mL) at 37 °C in a humidified atmosphere with 5% CO_2_. All cell lines were authenticated through short tandem repeat (STR) profiling conducted by the cell line characterization core (Genetic Testing Biotechnology Corporation, Suzhou, China); the report was attached in the Supplementary Materials Section S5.

### 2.3. Western Blot Analysis

Protein lysates were prepared using RIPA buffer supplemented with 1% Halt protease and phosphatase inhibitor cocktail (Thermo, Waltham, MA, USA, Cat. No.: 78442) on ice, followed by ice-bathed ultrasonication (SCIENTZ08-II, SCIENTZ, Ningbo, China) and centrifugation at 13,000× *g* for 12 min at 4 °C. The supernatant was collected. Protein concentration of lysates was determined by BCA assay. The protein lysates and 5× loading buffer (NCM-bio, Suzhou, China, # WB2001) were mixed at a 4:1 ratio and then heat-treated at 75 °C for 10 min to obtain SDS-PAGE samples. Samples containing equal amounts of total protein were resolved by SDS-PAGE and transferred to PVDF membranes. After blocking with 5% skim milk (2 h), membranes were incubated overnight at 4 °C with primary antibodies (Table S2), followed by HRP-conjugated secondary antibodies (2 h, RT). Signals were detected by ECL (Tanon 4600, Shanghai, China) and quantified with ImageJ 1.54g software.

### 2.4. Cell Cycle Test

Cell cycle distribution was analyzed by propidium iodide (PI) staining and flow cytometry Analysis. At the time of the cell cycle test, the cell was digested with 0.25% trypsin (containing EDTA), and PBS-washed cells were fixed in 70% ethanol at 4 °C for at least 12 h. Fixed cells were centrifuged (1000× *g*, 5 min, 4 °C), washed with PBS, and resuspended in PI/RNase A staining buffer. Following 30 min incubation at 37 °C in the dark, cells were analyzed by flow cytometry (FACSCelesta, Becton, Dickinson and Company, Franklin Lakes, USA). Data was processed using ModFit LT 5.0.9 software.

### 2.5. EdU Experiments

Cell proliferation was assessed by EdU assay. Cells were first incubated with 10 μM EdU (in complete medium) for 1–4 h (2 h for PC9, 4 h for HCC827) to incorporate EdU. After washing with PBS, the cells were fixed with 4% paraformaldehyde for 30 min and further permeabilized with 0.3% Triton X-100 for 30 min, followed by PBS washing. A click chemistry reaction was performed using the BeyoClick™ EdU Cell Proliferation Kit with AF594 (Beyotime, Shanghai, China) to conjugate the Alexa Fluor 594 fluorophore to EdU. The click reaction solution (containing Alexa Fluor 594-Azide, CuSO_4_, and other necessary components) was freshly prepared according to the manufacturer’s instructions and added to the fixed cells to initiate the click reaction. The reaction was conducted in the dark at room temperature for 30 min. Nuclei were counterstained with Hoechst 33342. Before observation, cells were washed three times with PBS. Fluorescence images were acquired using a CKX53 fluorescence microscope (Olympus, Tokyo, Japan).

### 2.6. Bim BH3 Transfection

Bim BH3, a 20-residue peptide containing the BH3 domain from the BH3-only protein Bim, was purchased from MCE (Monmouth Junction, NJ, USA, Catalog HY-P1527). The protein transfection reagent was purchased from MCE (Monmouth Junction, NJ, USA, Cat. No.: HY-K2016) to transfect the peptide into the cell. Briefly, Bim BH3 was dissolved in serum-free RPMI1640. The protein transfection reagent was then added and gently mixed. The mixture was incubated at room temperature for 20 min and further diluted with serum-free RPMI1640 to make the transfection working medium. Before the transfection, the cells were washed three times to completely remove the serum, and the transfection working medium was then added to the cells for transfection. The final concentration of Bim BH3 is 20 μM for MTT test and 34.1 μM for apoptosis rate and apoptosis-associated proteins determination.

### 2.7. siRNA Transfection

Cells were seeded in 96-well plates at a density of 5000 cells/well, and 2 d were allowed to adhere. For cell transfection, 30 or 90 nM siRNA was transfected into HCC827 cells using RNAimax (Invitrogen, Carlsbad, CA, USA), with the transfection mixture prepared in Opti-MEM. The siRNA sequences used in this study are listed in Table S12. The silencing efficacy of siRad51 and siTS are presented in Figure S6 and Table S13.

### 2.8. Cell Apoptosis Analysis

Apoptosis rate was quantified by Annexin V-FITC/PI staining using a BD FACSCelesta flow cytometer (BD Biosciences, Becton, Dickinson and Company, Franklin Lakes, NJ, USA). Cells were washed with PBS, dissociated with Accutase (Invitrogen, Carlsbad, CA, USA), and resuspended in staining buffer according to manufacturer’s protocol. After 10 min incubation in the dark at room temperature, samples were immediately analyzed by flow cytometry. Data were processed using FlowJo 10.8.1 software (FlowJo LLC, Ashland, OR, USA).

### 2.9. Cell Viability and Inhibition Assay

**Figure 1:**
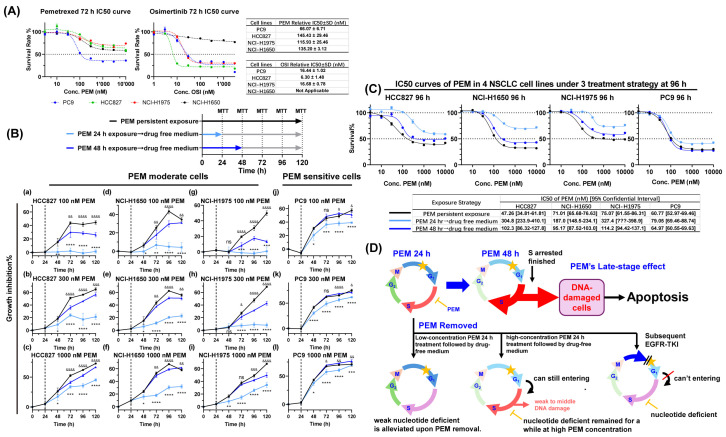
**The late-phase inhibition effect of PEM depends on the efficient PEM exposure time**. (**A**) 72 h IC50 curves of PEM and OSI in four NSCLC cell lines (*n* = 6). (**B**) As shown in the top schematic diagram, the inhibition rates of 4 NSCLC cells at various time points under following conditions: continuous exposure to 100, 300, and 1000 nM PEM or exposed to 100, 300, and 1000 nM PEM for 24 or 48 h, followed by drug removal and continued culture in drug-free medium (*n* = 6). (**C**) As illustrated in the schematic diagram from Panel B, the IC50 curve of PEM under three exposure schedules was analyzed at 96 h (*n* = 6). (**D**) Schematic diagram elucidating the differences among PEM exposure for 24 h, PEM exposure for 48 h, low-concentration PEM exposure for 24 h followed by drug withdrawal, high-concentration PEM exposure for 24 h followed by drug withdrawal, and high-concentration PEM exposure for 24 h followed by EGFR-TKI treatment. All data were presented as mean ± SD, *ns* not significant, * *p* < 0.05, ** *p* < 0.01, *** *p* < 0.001, and **** *p* < 0.0001 when comparing 48 h PEM exposure group to 24 h PEM exposure group; & *p* < 0.05, && *p* < 0.01, &&& *p* < 0.001, and &&&& *p* < 0.0001 when comparing 48 h PEM exposure group to persistent PEM exposure group. ???: the lower boundary of 95% Confidential interval cannot be evaluated.

Cell viability was measured using the MTT assay. Briefly, cells were seeded into 96 well plates in 100 μL of complete medium at a density of 5000 cells/well (2500 cells/well for experiments in Figure 1 and Figure S2). After different treatments and at the time of the MTT test, the old medium was discarded, cells were washed with PBS, and serum-free RPMI-1640 medium containing 0.5 mg/mL MTT was added. The cells were incubated for 1–2 h, the supernatant was discarded, the purple formazan precipitate was shaken dissolved in 100 μL DMSO, and absorbance was read at 570 nm using a SpectraMax 190 microplate reader (Molecular Devices, San Jose, CA, USA). The survival% and growth inhibition% were calculated using the following equation:

(1) $$\mathrm{Survival}\%=\frac{OD_{\mathrm{test}}-OD_{\mathrm{blank}}}{OD_{\mathrm{control}}-OD_{\mathrm{blank}}}\times 100\%$$

(2) Growth inhibition%=100−Survival%

### 2.10. Immunofluorescence

Cells were fixed with 4% paraformaldehyde, permeabilized in 0.3% Triton X-100, and blocked with 10% goat serum. After overnight incubation with primary antibody at 4 °C, samples were washed with PBS and incubated with Alexa Fluor-488/594-conjugated secondary antibodies for 2 h at room temperature. Nuclei were counterstained with Hoechst 33342 following secondary antibody removal and PBS washes. Fluorescence images were acquired using a CKX53 microscope (Olympus, Tokyo, Japan).

### 2.11. In Vivo Anti-Cancer Efficacy Study in HCC827 Xenograft Mice

The in vivo study strictly adhered to the protocols approved by the Animal Ethics Committee of China Pharmaceutical University (Approval No. YSL-202504062). Healthy female Balb/c Nude mice, aged 6–8 weeks and weighing 18–22 g, were supplied by Hangzhou Ziyuan Laboratory Animal Co., Ltd. (Hangzhou, China) (Certificate No. 20250511Abzz01050000024). The mice were housed under constant humidity (50 ± 10%), constant temperature (22 ± 2 °C), and a normal day-night cycle, with ad libitum access to food and water.

HCC827 tumor cells (1 × 10^7^ cells/mouse) were subcutaneously inoculated into the right flank of the Balb/c Nude mice. Prior to inoculation, cells were resuspended in serum-free medium-high concentration matrigel (1:1, *v*/*v*) to a density of 1 × 10^8^/mL, with 100 μL of the suspension injected per mouse. When the average tumor volume reached 200 mm^3^, tumor-bearing mice were randomly grouped and subjected to drug treatment (dosing regimen illustrated in Figure 10A): PEM was dissolved in saline and administered intraperitoneally at 35 mg/kg per administration (three times daily at 4 h intervals, total daily dose 105 mg/kg). OSI was dissolved in 1% Tween-80 and orally administered via gavage at 1 mg/kg/day. Tumor length (a) and width (b), as well as mouse body weight, were measured every 3 days after the start of the drug treatment. Tumor volume was calculated according to the following equation:

(3) $$V_{\mathrm{tumor}}=\frac{\pi }{6}ab^{2}$$

### 2.12. TUNEL and Immunohistochemical Staining of Tumor Tissues

After dissecting the tumor tissue, it was fixed in 4% paraformaldehyde solution for at least 48 h, followed by paraffin embedding and sectioning. The TUNEL and immunohistochemical staining were performed by Nanjing JinYiBai Biological Technology Co., Ltd. (Nanjing, China). Briefly, the tumor sections were deparaffinized, blocked, and subjected to antigen retrieval. For immunohistochemical staining, the section was incubated with primary antibodies overnight at 4 °C and then washed and incubated with HRP-linked secondary antibody at room temperature for 30 min. Chromogenic detection was achieved by incubating the sections with DAB substrate, and nuclei were counterstained with hematoxylin. For TUNEL staining, after digestion with proteinase K, the sections were incubated with TUNEL reaction mixture (prepared following the instruction of the manufacturer) at 37 °C in the dark for 1 h. Following three washes with PBS, the nuclei were counterstained with DAPI.

### 2.13. Statistical Analysis

All data are expressed as mean ± SD. Statistical analyses were performed using GraphPad Prism 8.0 (GraphPad Software, San Diego, CA, USA). Differences between two groups were assessed by unpaired Student’s *t*-test, while comparisons among multiple groups used analysis of variance (ANOVA). Nonlinear regression analyses (four-parameter variable slope model for agonist/inhibitor vs. response) were implemented in GraphPad Prism 8.0. The number of experimental replicates (*n*) and significance levels are described in the corresponding figure legends.

## 3. Results

### 3.1. The Late-Phase Inhibition of Pemetrexed Depending on the Efficient Pemetrexed Exposure Time

The 72 h IC_50_ curves for PEM and OSI across four cell lines are shown in Figure 1A. Based on PEM sensitivity, the four cell lines can be classified as PEM-sensitive PC9 and PEM-moderately sensitive NCI-H1975, NCI-H1650, and HCC827. Based on OSI sensitivity, the four cell lines can be classified as OSI-sensitive PC9, HCC827, NCI-H1975, and OSI-insensitive NCI-H1650. The expression levels of PEM and OSI pharmacodynamic-associated proteins (TS and Rad51 for PEM, EGFR, Akt, ERK1/2, and Bim for OSI) in the four NSCLC cell lines, along with their detailed genetic background characteristics, are presented in Figure S1 and Table S3 [25,26,27,28,29,30], respectively.

While administration sequence is a research focus [18,19], the impact of administration schedule on PEM’s baseline late-phase efficacy is often overlooked. If PEM fails to mediate a significant late-phase inhibitory effect, the synergistic efficacy of OSI with PEM could remain limited. PEM is clinically administered once every 21 days. Plasma concentrations of PEM sustained above efficient levels (e.g., ≥0.15 μM, reference to the PEM IC50 curve in the four NSCLC cells) persist for 48 h at most [31]. Based on the hypothesis from previous studies [19,21], OSI may antagonize PEM’s S-phase arrest through its G1-phase arresting effect. This implies that premature administration of OSI could render residual PEM in plasma ineffective, as OSI interference prevents PEM from exerting early-phase cytotoxic activity, indicating that the effective exposure time of PEM is modulated by both pharmacokinetic of PEM and the administration regimen of OSI. The sequential interval essentially represents the effective exposure time of PEM within the sequential PEM → OSI strategy.

Although the 24 h interval PEM → EGFR-TKI regimen is commonly used in previous studies [18,19], its sufficiency to elicit a strong late-phase effect to be synergized by OSI is unclear. To address this, as indicated in Figure 1B, we systematically analyzed the relationship between PEM exposure duration and its late-phase inhibition efficacy, with the aim to explore how sequential intervals modulate the intensity of PEM’s late-phase effects. The inhibition rate-time course profiles of four NSCLC cell lines were tested following persistent exposure, 24 h exposure, and 48 h exposure to PEM at concentrations of 100 nM, 300 nM, and 1000 nM. In PEM-sensitive PC9 cells, 24 h exposure achieved inhibition rates comparable to persistent exposure to PEM (Figure 1B), whereas in PEM moderate cells (HCC827/NCI-H1975/NCI-H1650), 24 h exposure produced only minimal effects, no late-phase inhibition at 100 nM, and weak late-phase effects at 300 nM. By contrast, 48 h PEM exposure generates the late-phase inhibition near the inhibition rate under PEM persistent exposure (Figure 1B). The 96 h IC_50_ curves further confirmed this conclusion (Figure 1C); in PEM moderate cells, 48 h exposure exhibited slightly higher IC_50_ than that in persistent PEM exposure, while 24 h exposure showed significantly higher IC_50_ (Figure 1C), indicating that inadequate exposure time drastically diminishes efficacy. Whereas in PEM-sensitive PC9 cells, the three-exposure schedule has a similar IC_50_ (Figure 1C, 60.77, 79.06, and 64.97 nM, respectively).

Dynamic analysis of cell cycle and DNA damage level (indicated by γ-H2AX and p-P53) revealed critical time windows for PEM effects. Taking HCC827 cells as an example, 1 μM PEM exposure for 24 h induced only mild S-phase arrest and DNA damage. However, during the 24 to 48 h period, the degree of S-phase arrest and DNA damage markers surged dramatically (Figures 4I and 6A). This indicates that PEM’s effects transitioned from initial accumulation to a rapidly accelerating phase during this period, demonstrating that the late-phase effect has a time-dependent threshold, where PEM-moderate cells require sufficient exposure duration (e.g., 48 h) to trigger significant damage accumulation.

Notably, as illustrated in Figure 1D, while 1000 nM PEM for 24 h achieved about half-inhibition of persistent exposure, subsequent EGFR-TKI differed fundamentally from withdrawal. Withdrawal allows ongoing S-phase arrest/DNA damage, whereas EGFR-TKI administration immediately arrests non-S-phase cells in G1, shielding them from PEM’s effects and weakening late-phase inhibition. This suggests premature EGFR-TKI interrupts PEM’s early-phase effect accumulation.

### 3.2. The Synergistic Effect Between PEM and OSI Depends on Both the Sequence Strategy and Sequential Interval

**Figure 2:**
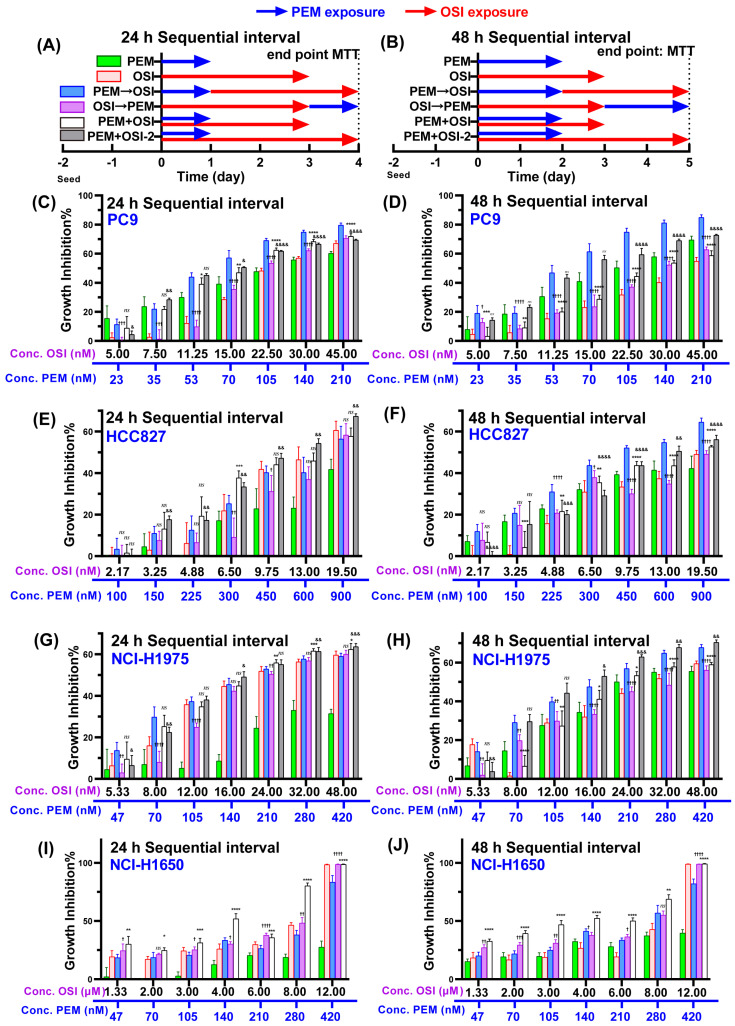
**The synergistic effect of PEM and OSI depends on both the administration sequence and sequential interval**. (**A**,**B**) Schematic representation of the evaluated sequential strategies and sequential interval. (**C**–**J**) Growth-inhibition rate of the four NSCLC cell lines treated with different sequence strategies and sequential intervals; the legend for each column was presented in Panel (**A**,**B**); the sequential interval used were presented at the title of each panel. The concentration settings for each PEM–OSI combination correspond to the concentration axes of PEM and OSI beneath each panel. All data were presented as mean ± SD (*n* = 6), *ns* not significant, * *p* < 0.05, ** *p* < 0.01, *** *p* < 0.001, and **** *p* < 0.0001 when comparing PEM → OSI group to PEM + OSI group, † *p* < 0.05, †† *p* < 0.01, ††† *p* < 0.001, and †††† *p* < 0.0001 when comparing PEM → OSI group to OSI → PEM group, & *p* < 0.05, && *p* < 0.01, &&& *p* < 0.001, and &&&& *p* < 0.0001 when comparing PEM → OSI group to PEM + OSI-2 group.

Figure 1 demonstrates the significant differences in late-phase inhibitory effects between 24 h and 48 h PEM exposure in PEM-moderate NSCLC cells. According to our theoretical framework, the intensity of PEM’s late-phase inhibitory effects governs OSI’s capacity to induce pro-apoptosis during this phase, consequently determining the magnitude of synergistic potency (as summarized in Figure 7J). Thus, we hypothesized that immediate EGFR-TKI combination after 24 h PEM exposure might arrest tumor cells not yet entering S-phase at G1-phase, thereby attenuating late-phase effects and losing synergistic efficacy (Figure 1D), while a 48 h PEM exposure achieves a late-phase inhibition rate comparable to that of continuous PEM exposure. At this point, PEM-induced early S-phase arrest and DNA damage accumulation are complete; thus, subsequent administration of OSI is sufficient to elicit robust synergistic effects. To validate our hypothesis and theoretical framework, we systematically evaluated the inhibitory effects under different sequence strategies with a 24 and 48 h sequential interval, as illustrated in Figure 2A,B. Quantitative synergy metrics analyses were performed using the Bliss score method, and Bliss scores under each sequencing strategy with different sequential intervals are listed in Tables S4–S11.

Unlike PEM (whose effective exposure duration is limited by the sequential strategy and sequential interval, as illustrated in Figure 1), OSI is administered clinically in a once-daily, continuous-dosing regimen (e.g., in a 28 d clinical PEM → almonertinib sequential combination strategy, PEM is administered on Day 1, followed by continuous almonertinib administration for three weeks starting from Day 8 [18]). Therefore, regardless of the sequential strategy implemented or the sequential interval set, OSI consistently achieves persistent exposure state through continuous drug administration. Given that there is currently no reported evidence indicating that PEM-mediated S-phase arrest antagonizes the effects of OSI, OSI can achieve steady-state inhibition through its sustained and effective exposure across different sequential combination strategies. We examined the relationship between OSI exposure duration and inhibition rate, revealing that OSI-mediated inhibition reached a plateau after 72 h of exposure (Figure S2). Therefore, we selected a 72 h OSI exposure period in the experimental design of Figure 2 to simulate its steady-state inhibitory effects in clinical practice.

Although concurrent PEM + OSI exhibited optimal inhibition in NCI-H1650 cells (Figure 2I,J and Tables S10 and S11), this occurred at OSI concentrations far exceeding clinically achievable plasma levels (clinical-free OSI concentration fluctuated between 10–50 nM, Supplementary Material Section S4) [32,33,34,35], limiting its relevance. Therefore, we focused subsequent analyses on three OSI-sensitive cell lines (PC9, HCC827, and NCI-H1975) with greater clinical translatability.

The 24 h interval sequential PEM → OSI strategy demonstrated synergy only in PEM-sensitive PC9 cells (Figure 2C and Table S4). This experimental observation aligns with our theoretical framework. As demonstrated in Figure 1B, in PEM-sensitive PC9 cells, 24 h PEM exposure induced potent late-phase inhibitory effects comparable to persistent PEM treatment. This indicates that 24 h exposure sufficed to complete S-phase arrest and DNA damage accumulation, thereby enabling subsequent synergy with OSI in PC9. Conversely, in the other three moderately PEM-sensitive cell lines, 24 h PEM exposure failed to induce pronounced late-phase inhibitory effects (Figure 1B), resulting in limited OSI synergy (Figure 2E,G and Tables S6 and S8). Notably, when employing the 48 h interval sequential PEM → OSI strategy, all three OSI-sensitive cell lines exhibited synergistic effects, with inhibitory efficacy surpassing monotherapy and other sequential strategies at equivalent total drug exposure (Figure 2D,F,H and Tables S5, S7 and S9). In EGFR/Bim-high-expressing PC9 and HCC827 cells (Figure S1), the sequential PEM → OSI regimen (Figure 2D,F and Tables S5 and S7) demonstrated stronger inhibition than the PEM + OSI-2 strategy (which has a higher total drug exposure), whereas in EGFR and Bim-low-expressing NCI-H1975 cells, the inhibition effect was comparable to the PEM + OSI-2 strategy (Figure 2H). Although PC9 cells already demonstrated synergistic effects with the 24 h interval sequential PEM → OSI strategy, the 48 h interval further enhanced synergistic potency (Figure 2C,D and Tables S4 and S5). The Bliss score increased from −0.022 at the 24 h sequential interval scenario to 0.053 at the 48 h sequential interval scenario.

Results from Figure 1 and Figure 2 demonstrate that the sequential PEM → OSI strategy achieves significant synergy (Figure 2C,D,F,H) only when PEM exposure duration suffices to induce potent late-phase inhibitory effects (Figure 1B). Conversely, when the effective PEM exposure time is insufficient to elicit a strong late-phase inhibitory effect (Figure 1B), lack of synergy, even with the application of the sequential PEM → OSI strategy (Figure 2E,G). These results validated the initial hypothesis: In PEM-moderate cells, insufficient S-phase arrest and DNA damage induced by 24 h exposure, subsequent with premature EGFR-TKI administration, caused G1-phase arrest, impairing late-effect generation and disrupting synergy. Extending PEM exposure to 48 h sufficiently accumulated S-phase arrest and DNA damage, triggering apoptosis signal at 60 h (Figure 7A), establishing the foundation for subsequent EGFR-TKI-mediated synergistic effects, ultimately achieving significant synergy in OSI-sensitive cells (Figure 7J).

### 3.3. OSI Promotes PEM-Induced Pre-Apoptosis Under 48 h Interval Sequential PEM → OSI Strategy

PEM induces S-phase arrest, triggering replication fork stalling and DNA damage, thereby activating the mitochondrial apoptosis pathway [36]. Based on our pilot experiments, OSI downregulates homologous recombination repair proteins and upregulates the pro-apoptotic protein Bim, which theoretically enables strong synergism with the late-stage effects of PEM. Additionally, Icotinib has been reported to enhance PEM-induced pro-apoptotic priming [19].

**Figure 3:**
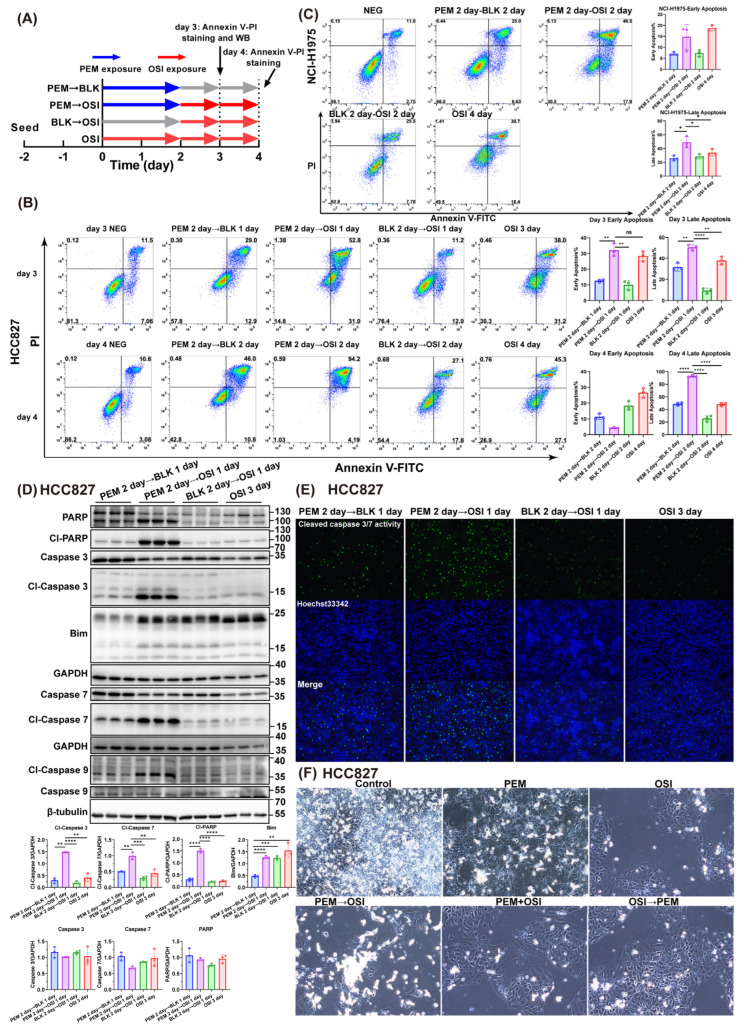
**OSI promotes PEM-induced pre-apoptosis**. (**A**) Schematic presentation of dosing schedule for panel (**B**–**D**). (**B**) HCC827 was treated with 1 μm PEM and 20 nM OSI with dosing schedule as indicated in Panel A; the apoptosis rate was determined at Day 3 and Day 4 by Annexin V-PI double-staining method (*n* = 3). (**C**) NCI-H1975 was treated with 1 μm PEM and 50 nM OSI with dosing schedule as indicated in Panel A; the apoptosis rate was determined at Day 4 by Annexin V-PI double-staining method (*n* = 3). (**D**) The apoptosis-associated proteins of HCC827 on Day 3, experimental conditions, were same as Panel B (*n* = 3). (**E**) Fluorescence images of Caspase 3 activity, with green indicating Caspase 3 activity and blue representing Hoechst 33342. HCC827 were treated with 1 μm PEM and 50 nM OSI, with dosing schedule as indicated in Panel A, and the imaging was at Day 3 (*n* = 3). (**F**) Bright-field images of cell morphology for HCC827 following treatment indicated in Figure 2B at the endpoint (the concentrations of PEM and OSI were 1 μM and 50 nM, respectively) (*n* = 3). All data were presented as mean ± SD, *ns* not significant, * *p* < 0.05, ** *p* < 0.01, *** *p* < 0.001, and **** *p* < 0.0001. NEG (negative control): drug-free treatment with freshly seeded cells at high viability; BLK (blank control): drug-free medium or vehicle treatment.

To validate OSI’s pro-apoptotic synergy with PEM’s late-phase effects, we employed the treatment protocol in Figure 3A. In HCC827 cells, we assessed apoptosis induction by sequential PEM → OSI administration through flow cytometry-based apoptosis quantification, apoptosis-associated proteins, and Caspase 3 activity fluorescence imaging, as well as bright-field microscopy (Figure 3). Meanwhile, the apoptosis rate detected by flow cytometry in the NCI-H1975 cell line was also assessed (Figure 3C).

The results demonstrated that 48 h interval sequential PEM → OSI treatment significantly activated the apoptotic signaling pathway in HCC827 cells, with significant upregulation of Cl-PARP, Cl-Caspase 3, Cl-Caspase 7 and Cl-Caspase 9 (Figure 3D). Strikingly, the upregulation of Bim expression was observed only in groups that had been treated with OSI and was not observed in the PEM → BLK (BLK represent Blank control, representing drug-free medium or vehicle treatment in this study) group, indicating that Bim upregulation is dependent on OSI exposure (Figure 3D). The trend in apoptosis rates measured by flow cytometry (Figure 3B) mirrored these findings, with sequential PEM → OSI treatment demonstrating significantly elevated apoptosis on Day 4. The Cl-Caspase 3 activity fluorescence micrographs and cytomorphological alterations (Figure 3E,F) further corroborated this conclusion. Similar trends were observed in NCI-H1975 cells, but their pro-apoptotic response was less pronounced than in HCC827 cells (Figure 3C).

### 3.4. OSI-Mediated G1 Arrest Effect Attenuates the Cytotoxicity of Pemetrexed When OSI Was Administered Concurrent with PEM or Prior to PEM

**Figure 4:**
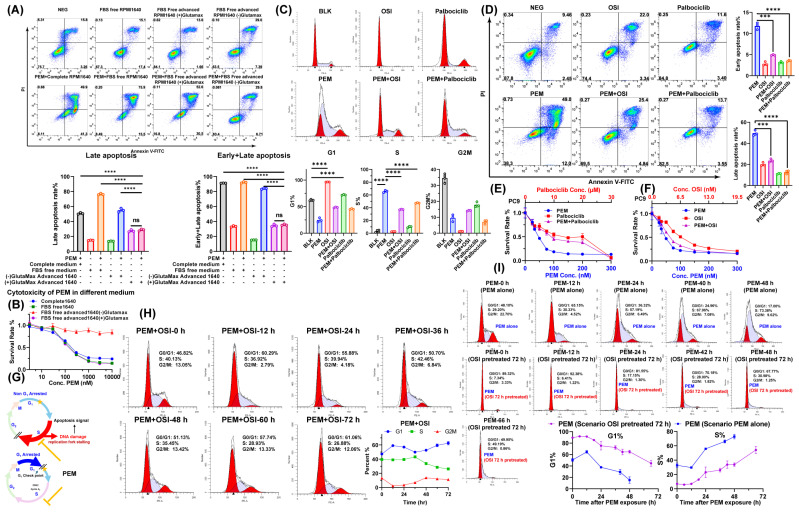
**OSI-mediated G1 arrest effect attenuates the cytotoxicity of pemetrexed when OSI was administered concurrently with PEM or prior to PEM**. (**A**,**B**) Panel (**A**) shows the apoptosis rate of PC9 cells treated with 300 nM PEM for 36 h under the following conditions: complete culture medium, FBS-free RPMI-1640, FBS-free Advanced RPMI 1640 supplemented with GlutaMax, and FBS-free Advanced RPMI 1640 without GlutaMax (*n* = 3). Panel (**B**) shows the survival rate concentration curve of PC9 cells under the same conditions after 72 h of treatment (*n* = 6). (**C**) PC9 cells were treated with BLK, 20 nM OSI, or 10 μM palbociclib, alone or with 300 nM PEM for 36 h, and the cell cycle analysis was performed via flow cytometry (*n* = 3). (**D**) The apoptosis rate of PC9 cells exposed to either 100 nM PEM alone or co-treated with 20 nM OSI and 10 µM palbociclib for 36 h (*n* = 3). (**E**,**F**) The survival–concentration curve of cells co-treated with higher concentrations of PEM and lower concentrations of OSI and palbociclib for 72 h; the combinatorial concentrations are as indicated on the axes (*n* = 6). (**G**) Under G1 phase arrest, the cell will not enter the S-phase, nor will it incur DNA damage caused by replication fork stalling resulting from S-phase arrest. (**H**) HCC827 cells were co-exposed with 50 nM OSI and 1 μM PEM for 72 h; cell cycle at each time point was analyzed by flow cytometry (*n* = 3). (**I**) HCC827 cells were pretreated with 50 nM OSI for 72 h and then continued to be cultured in RPMI-1640 medium containing 20% FBS supplemented with 1 µM PEM; cell cycle at each time point was analyzed by flow cytometry. Concurrently, the cell cycle of HCC827 cells without OSI pretreatment was also monitored. The rate of S-phase arrest was compared between the group pretreated with OSI for 72 h and the untreated group (*n* = 3); all data were presented as mean ± SD, *ns* not significant, *** *p* < 0.001, and **** *p* < 0.0001. NEG (negative control): drug-free treatment with freshly seeded cells at high viability; BLK (blank control): drug-free medium or vehicle treatment.

Mechanistically, PEM exerts cytotoxicity by arresting proliferating cells in S-phase, causing replication fork stalling and further inducing DNA damage and apoptosis [36]. This cytotoxicity may be strictly dependent on cells entering a proliferative state [36]. To verify this dependency, we selected the PEM-sensitive PC9 cell as a model to compare PEM toxicity under different culture conditions. Tested conditions included serum-free RPMI1640 (simulating low-growth-factor conditions), GlutaMax-free Advanced RPMI1640 (This medium lacks glutamine, which is essential for cell proliferation, to model a completely non-proliferative environment), and GlutaMax-containing Advanced RPMI1640 as control (Figure 4A,B). Results demonstrated that the low-growth-factor environment did not affect PEM toxicity. Only under complete proliferative arrest (glutamine-deprivation condition) was PEM toxicity significantly attenuated (Figure 4A,B). This definitively confirms that PEM cytotoxicity is proliferation-dependent, but antagonizing PEM cytotoxicity requires sufficiently potent proliferation suppression.

OSI suppresses CDK4/6 activity by downregulating Cyclin D1 and upregulation of p27 [37], whereas the CDK4/6 inhibitor palbociclib directly inhibits CDK4/6 [38]. Both agents induce G1-phase arrest by impairing Cyclin D1-CDK4/6 complex activity, but whether this G1 arrest translates to sufficiently potent proliferation suppression requires further evaluation. To validate the impact of this Cyclin D1–CDK4/6 complex-targeted G1 arrest on PEM cytotoxicity, we used low concentrations (proliferation-inhibiting concentrations) of OSI and the CDK4/6 inhibitor palbociclib in synchronous exposure with relatively high concentrations of PEM to assess their effects on the cell cycle, apoptosis rate, and inhibition rate. The results demonstrated that PEM exposure alone induced cell cycle arrest in S-phase (Figure 4C). When co-exposed with PEM, both OSI and palbociclib significantly attenuated PEM-induced S-phase arrest (Figure 4C). Compared with PEM monotherapy, synchronous combination of either OSI or palbociclib with PEM substantially diminished PEM-induced cytotoxic and apoptotic effects (Figure 4D). The concentration-response curves of high-concentration PEM combined with low-concentration OSI or palbociclib exhibited a marked antagonistic effect (Figure 4E,F), revealing that G1-phase arrest potently counteracts PEM-induced cytotoxicity, as illustrated in Figure 4G. This finding was also observed in γ-H2AX immunofluorescence images of HCC827 cells, where PEM induced potent DNA damage, but co-exposure with OSI attenuated the DNA-damaging efficacy of PEM (Figure S5A).

Subsequently, we analyzed the cell cycle kinetic profiles of HCC827 cells under two schedules: sequential OSI → PEM regimens and concurrent PEM+OSI regimens. When cells were treated with OSI alone, OSI totally arrested cells into G1 phase within 36 h (Figure S3), but in PEM + OSI regimens, OSI cannot instantaneously induce G1-phase arrest. A considerable proportion of cells remain in S-phase, with 30% still in S-phase at the 48 h time point (Figure 4H). This indicates that PEM may still be able to mediate partial S-phase arresting effects. In contrast, in the sequential OSI → PEM strategy, after 72 h of OSI pretreatment, the proportion of S-phase cells was reduced to 0. Furthermore, the accumulation rate of subsequent PEM-induced S-phase arrest was significantly decreased (Figure 4I). These results suggest that although the intensity of S-phase arrest induced by PEM in the concurrent combination strategy is less potent than in the sequential PEM → OSI strategy, its residual blocking effect can still trigger some late-stage cytotoxicity that can be enhanced by OSI. In contrast, the sequential OSI → PEM strategy exhibits the lowest level of S-phase arrest within 48 h of PEM exposure. This phenomenon is consistent with previously reported findings, where the concurrent PEM + OSI regimen demonstrated an additive effect, while the sequential OSI → PEM regimen exhibited an antagonistic effect [18,19,21].

### 3.5. The Washout Period for OSI-Mediated G1 Arrest Is No More than 48 h

A critical challenge in the PEM → EGFR-TKI sequential regimen lies in the requirement to suspend EGFR-TKI between treatment cycles to resolve G1 arrest and prevent its attenuation of subsequent PEM efficacy, as illustrated in Figure S4 [19]. As an irreversible EGFR inhibitor, OSI blocks kinase activity by binding to the Cys-797 residue of EGFR, and its effects diminish only upon tumor cell-mediated EGFR receptor resynthesis [39]. If OSI-induced G1 arrest persists excessively, sequential PEM → OSI therapy may lose its advantage due to potential disease progression during the treatment-free interval, rendering concurrent PEM + OSI combination clinically preferable (Figure S4).

**Figure 5:**
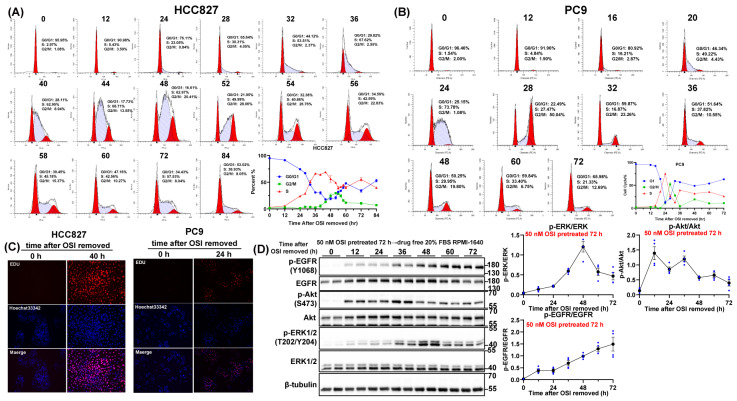
**The washout period for OSI-mediated G1 arrest is no more than 48 h**. (**A**–**C**) HCC827 and PC9 cells were pretreated with 50 nM or 40 nM OSI, respectively, for 72 h. After three PBS washes, cells were cultured in drug-free RPMI1640 medium supplemented with 20% FBS (HCC827) or 10% FBS (PC9); cell cycle at each time point was analyzed by flow cytometry. Concurrently (Panel (**C**)), EdU incorporation assays were performed immediately after OSI withdrawal (0 h) and at proliferation assessment endpoints (40 h for HCC827 and 24 h for PC9), followed by click chemistry-based EdU staining and fluorescence microscopy (*n* = 3). (**D**) HCC827 cells pretreated with 50 nM OSI for 72 h and washed 3 times, cultured in 20% FBS-containing medium for 72 h post-drug removal, and sampled every 12 h for EGFR signaling pathway analysis via WB assay (*n* = 4). All data were presented as mean ± SD.

To address this, we assessed the time required for G1 arrest to disappear post-OSI withdrawal in PC9 and HCC827, two NSCLC cell lines with high EGFR expression (Figure S1) and OSI sensitivity (Figure 1A). Cells were pretreated with 40 nM (PC9) or 50 nM (HCC827) OSI for 72 h, followed by dynamic monitoring of cell cycle progression after OSI removed. PC9 and HCC827 entered S-phase at about 24 and about 40 h post-OSI withdrawal (Figure 5A,B), respectively, and gradually advanced to M-phase. EdU assays confirmed proliferation resumption in PC9 and HCC827 within 24 and 40 h after OSI removal (Figure 5C). In HCC827, EGFR signaling recovered within 60 h post-OSI withdrawal, with earlier restoration of phosphorylated ERK1/2 and Akt levels (Figure 5D).

Supporting these findings, a PK/PD modeling study revealed EGFR turnover rates of 0.06, 0.02508, and 0.01809 h^−1^ in A431, PC9, and NCI-H1975 xenografts, corresponding to 50% EGFR recovery times of 11.55, 27.63, and 38.31 h [39]. This aligns with our observation that PC9 cells resumed proliferation within 24 h post-OSI withdrawal. This indicates that sequential PEM → OSI therapy does not necessitate prolonged drug-free intervals, thereby bolstering confidence in the clinical feasibility of the PEM → OSI regimen.

### 3.6. The Pharmacodynamic Interaction Between PEM-Triggered DNA Damage and OSI-Driven Rad51/TS Inhibition Under Different Sequencing Conditions

PEM causes replication fork stalling through S-phase arrest, which subsequently induces DNA damage (Figure S5A; the PEM-induced DNA damage level is attenuated by concurrent OSI) [36]. Homologous recombination repair (HRR) plays a critical role in DNA damage repair during the S/G2 phases, with Rad51 acting as the core factor in the HRR pathway [40]. Rad51 expression is regulated by the Ras/Raf/MEK/ERK1/2-Rad51 signaling axis (Figure 9D,F) [41,42]. Downregulation of Rad51 may enhance NSCLC cell sensitivity to PEM [43,44]. Moreover, OSI may downregulate Rad51 by suppressing ERK1/2 signaling (Figure 9D); this downregulation potentially enhances NSCLC cell sensitivity to PEM [43,44]. TS serves as a key predictive biomarker for PEM sensitivity. Multiple studies demonstrate that either low baseline TS levels or pharmacologically induced TS downregulation enhances cellular sensitivity to PEM [4,23,24,45]. Pilot experiments in this study revealed that subsequent OSI administration in the sequential PEM → OSI combination significantly downregulated both Rad51 and TS (Figure S5C,D). Thus, during combination treatment, OSI may enhance PEM-mediated DNA damage by suppressing Rad51-dependent DNA repair while also sensitizing cells to PEM’s late-phase effects through TS downregulation.

**Figure 6:**
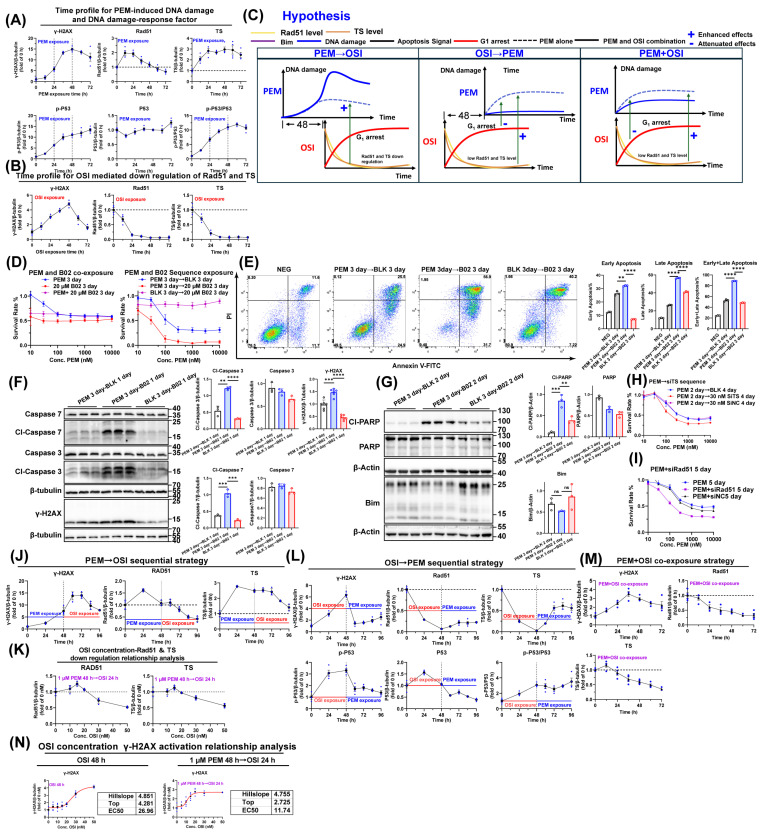
**The pharmacodynamic interaction between PEM-triggered DNA damage and OSI-driven Rad51/TS inhibition under different sequencing conditions**. (**A**) HCC827 cells were treated with 1 μM PEM, and the DNA damage-response proteins (γ-H2AX, p-P53, and P53), Rad51, and TS were analyzed by WB (*n* = 4). (**B**) HCC827 cells were treated with 20 nM OSI, and the DNA damage-response proteins (γ-H2AX, p-P53, and P53), Rad51, and TS were analyzed by WB (*n* = 4). (**C**) Schematic illustration of pharmacodynamic interactions between PEM-induced DNA damage and OSI-mediated downregulation of Rad51 and TS. (**D**) Concurrent exposed PEM with 20 μM B02 for 72 h shows no synergy in HCC827 (*n* = 6), but sequential PEM 72 h → 20 μM B02 strategy shows synergy (*n* = 6). (**E**–**G**) The apoptosis rate and apoptosis-associated proteins of HCC827 following indicated treatments (the concentrations of PEM and B02 were 1 μM and 20 μM, respectively) (*n* = 3). (**H**) HCC827 was exposed to PEM for 48 h, followed by BLK, 30 nM siTS, and 30 nM siNC treated for 96 h (*n* = 6). (**I**) PEM were co-exposed with 90 nM siRad51 for 5 days (*n* = 6). (**J**) HCC827 cells were first treated with 1 μM PEM for 48 h, followed by treatment with 50 nM OSI for 48 h. Rad51, TS, and γ-H2AX were analyzed by WB (*n* = 4). (**K**) The concentration-response curves of Rad51 and TS, HCC827 were pretreated with 1 μM PEM for 48 h, followed by OSI treatment at different concentrations for 24 h (*n* = 4). (**L**) HCC827 cells were first treated with 50 nM OSI for 48 h, followed by 1 μM PEM treated for 48 h. γ-H2AX, Rad51, TS, p-P53, and P53 were analyzed by WB (*n* = 4). (**M**) HCC827 cells were co-treated with 1 μM PEM and 50 nM OSI for 72 h. Rad51, TS, and γ-H2AX were analyzed by WB (*n* = 4). (**N**) The concentration-response curves of γ-H2AX in HCC827 cells under different treatment conditions: OSI treated at various concentrations for 48 h and 1 μM PEM treated for 48 h, followed by OSI treated at different concentrations for 24 h. All data were presented as mean ± SD, *ns* not significant, ** *p* < 0.01, *** *p* < 0.001, and **** *p* < 0.0001. NEG (negative control): drug-free treatment with freshly seeded cells at high viability; BLK (blank control): drug-free medium or vehicle treatment. All corresponding WB images for each panel in this figure were shown in Figure S7.

To validate the temporal overlap between PEM-induced DNA damage and OSI-mediated downregulation of Rad51/TS, we monitored kinetic profiles of DNA damage markers (γ-H2AX, p-P53, and p53) and Rad51/TS in HCC827 cells exposed to 1 μM PEM and 20 nM OSI (Figure 6A,B). PEM-induced DNA damage remained weak during 0–24 h, increased sharply from 24–48 h (Figure 6A), and reached peak level at 48 h. The p-P53 kinetics mirrored this pattern, suggesting 24–48 h represents the critical DNA damage accumulation window in HCC827 cells, which is a period that should not be interrupted by OSI-mediated G1 arrest effects. PEM-mediated DNA damage and one-carbon unit depletion upregulate Rad51 and TS (Figure 6A). OSI exposure alone reduced Rad51 and TS to extremely low levels within 24 h (Figure 6B). Notably, although OSI does not directly cause DNA damage mechanistically, it induced modest but detectable γ-H2AX upregulation peaking at 48 h (Figure 6B). This effect may be attributed to the accumulation of DNA damage resulting from OSI-mediated Rad51 inhibition. Based on their temporal dynamics, we propose the following hypothesis (Figure 6C): The sequential PEM → OSI regimen leverages peak PEM-induced DNA damage; subsequent OSI-mediated downregulation of Rad51 and TS impairs DNA repair, amplifying the DNA damage. Conversely, in both the concurrent PEM + OSI administration and the sequential OSI → PEM strategy, despite PEM exposure occurring in an environment of persistently suppressed Rad51 and TS levels, OSI-mediated G1 arrest significantly compromises S-phase arrest and DNA damage induction.

To validate that Rad51 inhibition synergizes with PEM’s late-phase effects to enhance DNA damage and exacerbate apoptosis, we first examined whether Rad51 suppression (via small-molecule inhibitor B02 [40]), Rad51 silencing, or TS silencing (siRad51 and siTS sequences were listed in Table S12; silencing efficacy validation was presented in Figure S6 and Table S13), could potentiate PEM’s late-phase efficacy. The results showed that concurrent administration of B02 and PEM for 72 h did not exhibit synergistic effects (Figure 6D), but sequential administration of B02 and PEM demonstrated significant synergistic effects (Figure 6D). Sequential PEM–B02 administration synergized with the late-phase effects of PEM, strongly enhancing apoptosis (Figure 6E–G). Furthermore, this promotion of apoptosis was independent of Bim (Figure 6G) and was accompanied by increased DNA damage level (Figure 6F). Additionally, silencing Rad51 also enhanced the inhibition rate of PEM (Figure 6I), and silencing TS with 30 nM siTS for 96 h after 48 h PEM exposure also demonstrated a certain degree of synergy (Figure 6H). This indicates that TS not only influences cellular sensitivity to PEM at the basal level, as previously reported in the literature [23,24], but also that drug-induced downregulation of TS can synergize with the late-phase effects of PEM, even after PEM removal.

We subsequently monitored kinetic profiles of DNA damage markers (γ-H2AX, p-P53, and p53) and Rad51/TS levels under different sequential strategies. Within the PEM → OSI sequence regimen, the addition of OSI downregulated the expression of both Rad51 and TS (Figure 6J), with the extent of downregulation exhibiting concentration dependency (Figure 6K). Specifically, treatment with 50 nM OSI for 24 h reduced the levels of Rad51 and TS to half of those observed in the untreated group (i.e., PEM 48 h → OSI 0 nM) (Figure 6K). In HCC827 cells, γ-H2AX levels reached their peak at 48 h following PEM exposure alone (Figure 6A). In contrast, under the sequential PEM → OSI regimen, the subsequent addition of OSI caused γ-H2AX levels to increase again and remain at high levels from 48 to 96 h. The peak of DNA damage occurred between 60 and 72 h, with an intensity twice that observed at 48 h (Figure 6J). This suggests that OSI likely enhances PEM-induced DNA damage by inhibiting Rad51. The PEM + OSI sequential regimen exhibited relatively weaker γ-H2AX levels (Figure 6M). In contrast, under the sequential OSI → PEM strategy, the initial OSI exposure induced a γ-H2AX peak; however, this peak disappeared following OSI removal. Subsequently, the following PEM treatment failed to induce significant DNA damage accumulation (Figure 6L), whereas this accumulation was clearly observed in PEM treatment without OSI pretreatment (Figure 6A). Concurrently, Rad51 and TS levels rebounded upon PEM exposure (Figure 6L) in OSI → PEM regimens.

Finally, we compared the concentration-response curves of OSI for inducing γ-H2AX in HCC827 cells under two treatment conditions: 1 μM PEM for 48 h followed by OSI for 24 h (PEM 48 h → OSI 24 h) versus OSI alone for 48 h (Figure 6N). The EC50 values were 11.74 nM and 26.96 nM for the sequential PEM → OSI treatment and OSI monotherapy, respectively. According to the nonlinear fitting parameter “top” (representing the fold-change of maximum effect relative to PEM 48 h → 0 nM OSI 24 h), OSI enhanced PEM-induced γ-H2AX levels by 2.725-fold (Figure 6N). The clinical-free plasma concentration of OSI fluctuates between 10–50 nM (Supplementary Material Section S4). In the sequential PEM → OSI therapy, due to the lower EC50 value of OSI, its fluctuating plasma concentration is consistently sufficient to effectively activate the DNA damage pathway. In contrast, during OSI monotherapy, only during the time window at high OSI-free concentration can DNA damage be effectively triggered.

In summary, only the 48 h interval sequential PEM → OSI strategy ultimately induced substantial DNA damage (Figure 6J,K). Although both PEM + OSI co-administration and OSI → PEM sequencing created low Rad51 and TS environments (Figure 6L,M), G1-phase arrest compromised the extent of S-phase arrest (Figure 4H,I), resulting in substantially reduced DNA damage (Figure 6L,M).

### 3.7. The Pharmacodynamic Interaction Between PEM-Induced Apoptotic Signaling and OSI-Induced Pro-Apoptotic Protein Bim Under Different Sequencing Conditions

**Figure 7:**
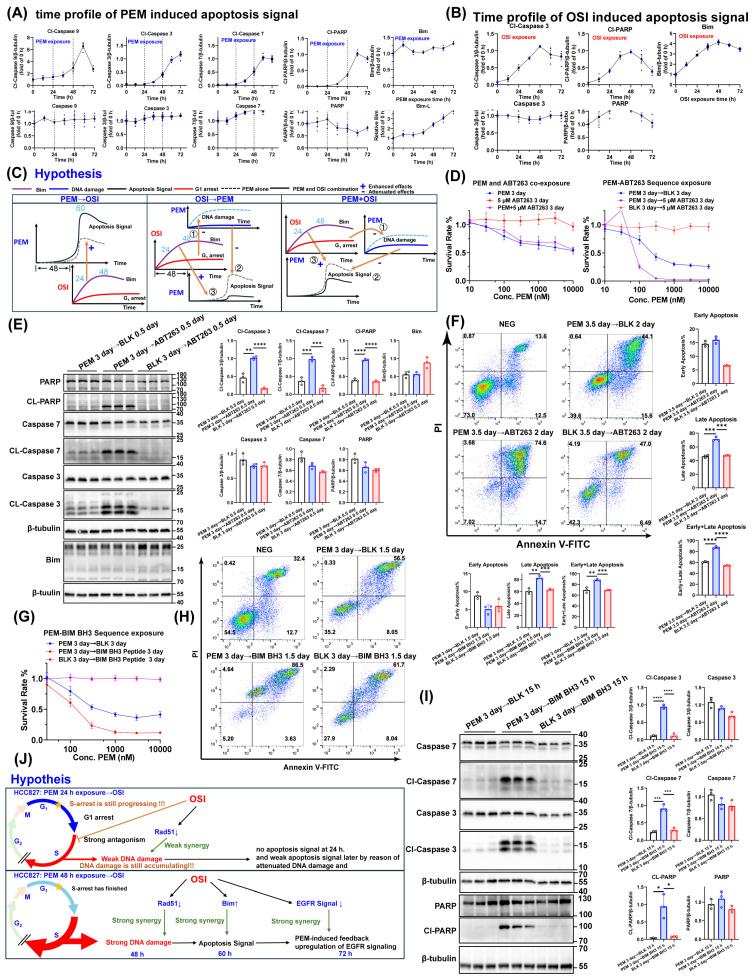
**The pharmacodynamic interaction between PEM-induced apoptotic signaling and OSI-induced pro-apoptotic protein Bim under different sequencing conditions**. (**A**) HCC827 cells were treated with 1 μM PEM for 72 h, and the apoptosis-associated proteins were monitored by WB (*n* = 4). (**B**) HCC827 cells were treated with 20 nM OSI for 72 h, and the apoptosis-associated proteins were monitored by WB (*n* = 4). (**C**) Schematic illustration of the pharmacodynamic interactions between PEM-induced apoptosis signal and OSI-induced BIM upregulation in different sequencing conditions. (**D**) PEM did not show synergy when co-exposed with 5 μM ABT263 for 72 h but shows strong synergy at PEM 72 h → 5 μM ABT 263 regimens (*n* = 6). (**E**,**F**) Apoptosis-associated proteins and apoptosis rate of HCC827 following indicated treatments (the concentrations of PEM and ABT263 were 1 μM and 5 μM, respectively) (*n* = 3). (**G**) PEM shows synergy at PEM 72 h → 20 μM BIM BH3 72 h regimens (transfected by protein transfection agent) (*n* = 6). (**H**,**I**) Apoptosis rate and apoptosis-associated proteins of HCC827 following indicated treatments (the concentrations of PEM and BIM BH3 were 1 μM and 34.1 μM, respectively) (*n* = 3). (**J**) Schematic of sequential interval modulation for optimal synergy in the PEM → OSI regimen. All data were presented as mean ± SD, * *p* < 0.05, ** *p* < 0.01, *** *p* < 0.001, and **** *p* < 0.0001. NEG (negative control): drug-free treatment with freshly seeded cells at high viability; BLK (blank control): drug-free medium or vehicle treatment. All corresponding WB images for each panel in this figure were shown in Figure S8.

Based on our observation that OSI potently enhances PEM-induced pro-apoptotic signaling (Figure 3) and the reported ability of Icotinib to promote PEM-induced apoptosis [19], we postulate that OSI amplifies PEM-triggered apoptosis not only by inhibiting Rad51-mediated homologous recombination repair but also by directly upregulating pro-apoptotic proteins. Pro-apoptotic protein Bim is the most crucial effector molecule in EGFR-TKI-induced apoptosis of NSCLC cells; resistance to many EGFR-TKIs is associated with reduced Bim, and upregulating Bim expression through pharmacological intervention can overcome OSI-acquired resistance [26,46,47]. We monitored the time course of apoptotic signaling in HCC827 cells exposed to 1 μM PEM and 20 nM OSI, respectively (Figure 7A,B). Apoptotic signaling was only initiated 48 h after PEM exposure alone, suggesting that administration of OSI before 48 h cannot enhance PEM-induced apoptotic signaling. The PEM-induced apoptotic signal intensified sharply during the 48–72 h period; however, no change was observed in Bim expression levels (Figure 7A). Interestingly, the peak of Bim signaling largely overlapped with the peak of OSI-mediated apoptotic signaling at 48 h post-OSI exposure (Figure 7B), suggesting a significant role for Bim in OSI-induced apoptosis. Furthermore, Bim expression was rapidly upregulated within 24 h of OSI exposure (Figure 7B). This indicates that in the 48 h interval sequential PEM → OSI strategy, the peak of PEM-induced apoptotic signaling and the peak of OSI-induced Bim upregulation can overlap temporally, providing the foundation for their synergistic effects. Based on this, we propose a hypothesis (Figure 7C). In the sequential PEM → OSI strategy, PEM administered in the early phase (unaffected by OSI) generates substantial apoptotic signaling. Subsequent OSI exposure then synergistically amplifies this signaling via Bim-mediated pro-apoptotic effects. In contrast, both the concurrent PEM + OSI strategy and the sequential OSI → PEM strategy fail to achieve effective synergy. Although early OSI exposure creates a high-Bim environment, Bim cannot cooperate with PEM during its initial exposure phase because OSI-induced G1 arrest compromises PEM-driven S-phase arrest and DNA damage, resulting in weak basal apoptotic signaling that cannot be potentiated by Bim (Figure 7C).

**Figure 8:**
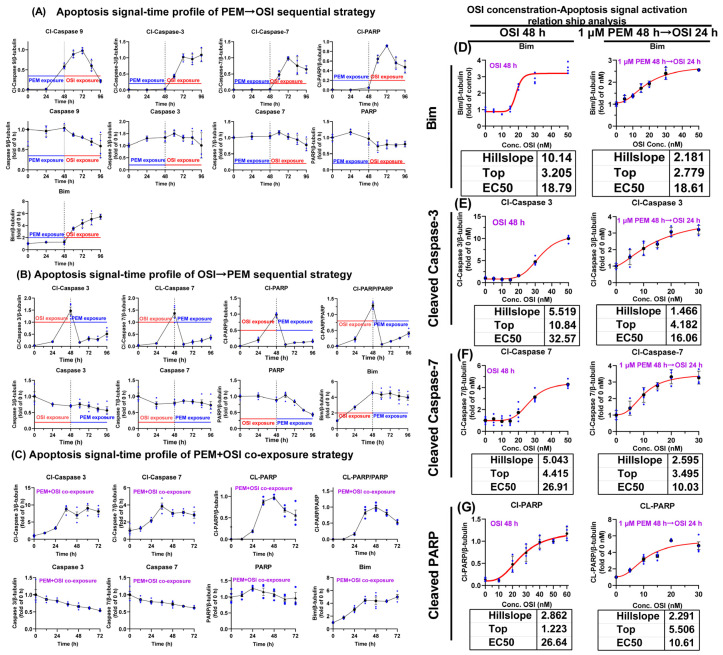
**Apoptotic-signaling kinetic profiles under different sequencing strategies and the concentration-response curves of OSI-induced activation of apoptosis-related proteins in sequential PEM → OSI combination versus OSI monotherapy**. (**A**) HCC827 cells were first treated with 1 μM PEM for 48 h, followed by 50 nM OSI treatment for 48 h. Apoptosis signaling was monitored by WB (*n* = 4). (**B**) HCC827 cells were first treated with 50 nM OSI for 48 h, followed by 1 μM PEM treatment for 48 h. Apoptosis signaling was monitored by WB (*n* = 4). (**C**) HCC827 cells were co-treated with 1 μM PEM and 50 nM OSI for 72 h. Apoptosis signaling was monitored by WB (*n* = 4). (**D**–**G**) Concentration-response curves of apoptosis-associated proteins in HCC827 cells under OSI 48 h (various concentrations) treatments or under 1 μM PEM 48 h → OSI 24 h (various concentrations) treatment (*n* = 4). All data were presented as mean ± SD. All corresponding WB images for each panel in this figure were shown in Figure S9.

To verify the pro-apoptotic role of Bim in the late-phase effects of PEM, we employed two approaches: the small-molecule Bim mimetic ABT263 (Figure 7D–F) [48] and the Bim BH3 peptide, a 20 aa peptide containing the core death domain of Bim protein (Figure 7G–I) [49,50]. We evaluated whether either agent exhibits sequential synergistic effects with PEM. The results demonstrated that both ABT263 and Bim BH3 potently enhanced the apoptotic rate and apoptotic signaling induced by the late-phase effects of PEM after only 12 h exposure (Figure 7D–I). This response closely resembled the immediate sharp increase in apoptotic signals observed following OSI exposure in the sequential PEM → OSI combination regimen (Figure 8A), indicating that upregulation of Bim may enhance PEM-induced pro-apoptotic signaling.

Subsequently, we monitored the time-course profile of apoptotic signaling across three sequential treatment regimens. In the sequential PEM → OSI strategy, administration of OSI was accompanied by significant upregulation of the Bim protein, driving a sharp intensification of apoptotic signaling (Figure 8A) and demonstrating Bim’s potent amplification effect on the PEM-induced apoptotic pathway. Before OSI administration, the clear detection of cleaved Caspase 9 (an early apoptosis marker) at 48 h post-PEM treatment (Figure 8A) demonstrates that the apoptotic program was initiating at this stage. This finding establishes the 48 h time point as the optimal window for OSI intervention (Figure 8A). Conversely, in the sequential OSI → PEM strategy, apoptotic signaling rapidly attenuated following OSI withdrawal, and subsequent PEM administration failed to effectively induce apoptotic signaling accumulation (Figure 8B). Apoptotic signaling induced by the concurrent PEM + OSI combination strategy was markedly weaker than that achieved with the sequential PEM → OSI regimen (Figure 8C).

Finally, we compared OSI’s activation of apoptosis-associated proteins between two regimens: (1) sequential PEM 48 h → OSI 24 h and (2) OSI alone 48 h. The EC50 curves for Bim activation (Figure 8D) were 18.61 nM under PEM 48 h → OSI 24 h treatment and 18.79 nM under OSI 48 h treatment alone. These nearly identical values indicate that PEM pretreatment does not affect OSI’s ability to activate Bim. The EC50 values for OSI-induced activation of Cl-PARP, Cl-Caspase 7, and Cl-Caspase 3 under OSI 48 h treatment were 26.64 nM, 32.57 nM, and 26.91 nM, respectively (Figure 8E–G), while corresponding values in the PEM 48 h → OSI 24 h regimen decreased to 10.61 nM, 16.06 nM, and 10.03 nM (Figure 8E–G). The significantly smaller EC50 values under the PEM 48 h → OSI 24 h sequential regimen indicated enhanced activation potency of OSI toward apoptosis-related proteins, demonstrating that minimal OSI concentrations can elicit maximal apoptotic signaling in this combination strategy. For OSI-induced activation of Cl-PARP, Cl-Caspase 7, and Cl-Caspase 3 in the PEM 48 h → OSI 24 h regimen, the nonlinear fitting parameter “top” (representing the fold-change of maximum effect relative to the PEM 48 h → 0 nM OSI 24 h) reached values of 5.506, 3.495, and 4.182, respectively (Figure 8E–G). This represents a 3.495- to 5.506-fold enhancement in maximal apoptotic signaling compared to PEM monotherapy (i.e., PEM 48 h → 0 nM OSI 24 h). The clinical-free plasma concentration of OSI fluctuates between 10–50 nM (Supplementary Material Section S4). In the sequential PEM → OSI therapy, due to the lower EC50 value of OSI, its fluctuating plasma concentration is consistently sufficient to effectively activate the apoptosis signal. In contrast, during OSI monotherapy, only during the time window at high OSI-free concentration can apoptosis signal be effectively triggered.

### 3.8. The Pharmacodynamic Interaction Between PEM-Induced Feedback EGFR Signaling and OSI-Mediated EGFR Signaling Suppression Under Different Sequencing Conditions

**Figure 9:**
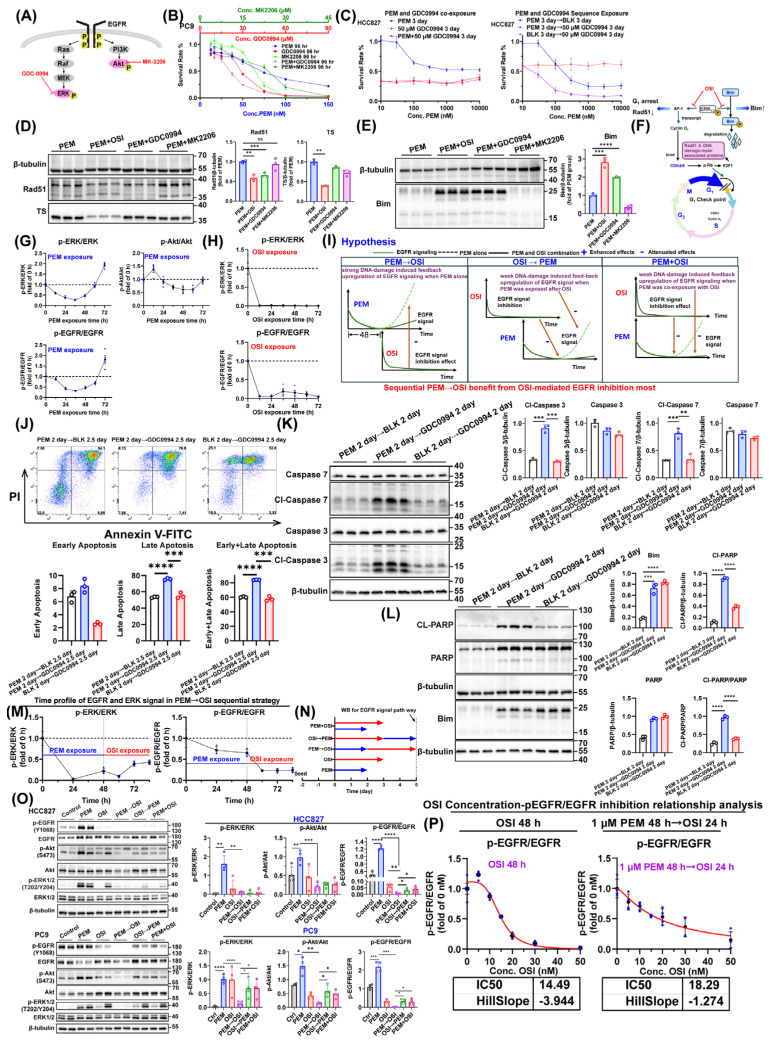
**The pharmacodynamic interaction between PEM-induced feedback EGFR signaling and OSI-mediated EGFR signaling suppression under different sequencing conditions**. (**A**) Schematic presentation of EGFR signaling pathway GDC0994 was used to block Ras/Raf/MEK/ERK signal, while MK2206 was used to block PI3K/PIP3/Akt signal. (**B**) Inhibition of ERK, instead of Akt, synergized with PEM in PC9 cells (*n* = 6). (**C**) Concurrent exposed PEM with 50 μM GDC0994 for 72 h shows no synergy in HCC827 (*n* = 6), but sequential PEM 72 h → 50 μM GDC0994 72 h regimens show synergy in HCC827 (*n* = 6). (**D**,**E**) Expression level of Rad51, TS, and Bim in HCC827 cells after 72 h exposure to either 1 μM PEM alone or 1 μM PEM combined with 20 nM OSI, 50 μM GDC0994, or 15 μM MK-2206 (*n* = 3). (**F**) Schematic illustration of ERK signaling blockade altering cell cycle progression and the protein expression of Rad51 and Bim. (**G**,**H**) The changes in EGFR signaling over time in HCC827 cells treated with 1 μM PEM or 50 nM OSI (*n* = 4). (**I**) Schematic illustration of the pharmacodynamic interaction between PEM-induced feedback EGFR signaling and OSI-mediated EGFR signaling inhibition under different sequential strategies. (**J**–**L**) Apoptosis rate and apoptosis-associated proteins of HCC827 following indicated treatments (the concentrations of PEM and GDC0994 were 1 μM and 50 μM, respectively) (*n* = 3). (**M**) HCC827 cells were first treated with 1 μM PEM for 48 h, followed by treatment with 50 nM OSI. EGFR signaling was monitored by WB (*n* = 4). (**N**) Scheme for evaluation EGFR signaling following different sequencing strategies. (**O**) EGFR signaling levels in HCC827 (1 μM PEM and 50 nM OSI) and PC9 (100 nM PEM and 40 nM OSI) cells at the experimental endpoint after treatment with the panel (N) indicated sequencing strategy (*n* = 4). (**P**) The concentration-response curves of pEGFR/EGFR in HCC827 cells under the following treatment conditions: OSI treatment at various concentrations for 48 h and pretreatment with 1 μM PEM for 48 h, followed by OSI treatment at different concentrations for 24 h (*n* = 4). All data were presented as mean ± SD, *ns* not-significant, * *p* < 0.05, ** *p* < 0.01, *** *p* < 0.001, and **** *p* < 0.0001; NEG (negative control): drug-free treatment with freshly seeded cells at high viability; BLK (blank control): drug-free medium or vehicle treatment. All corresponding WB images for each panel in this figure were shown in Figure S10.

The EGFR signal is transmitted downstream through two pathways: the Ras/Raf/MEK/ERK and PI3K/PIP3/Akt subpathways (Figure 9A) [51,52]. To clarify which downstream subpathway inhibition synergizes with PEM, we combined PEM with GDC-0994 [53] (an ERK1/2 inhibitor) and MK-2206 (an Akt inhibitor) [54] in PC9 cells. We found that PEM exhibited synergistic effects with GDC0994, but not with MK2206 (Figure 9B). In HCC827 cell line, while co-exposure to GDC0994 failed to synergize with PEM (Figure 9C), sequential PEM → GDC0994 treatment synergistically enhanced the late-phase effects of PEM (Figure 9C). This suggests that ERK1/2 inhibition plays a critical role in the synergy between EGFR-TKIs and the late-phase effects of PEM. Further studies in HCC827 cells showed that GDC0994-mediated inhibition of ERK1/2 not only downregulates Rad51 (Figure 9D) but also upregulates Bim (Figure 9E), whereas MK2206-mediated inhibition of Akt does not affect either protein. In contrast, neither GDC0994 nor MK2206 downregulated TS (Figure 9D,E). Building on the findings from this study with literature [32,37], we constructed a schematic diagram illustrating the proposed mechanism by which OSI synergizes with PEM through ERK1/2 inhibition (Figure 9F).

To clarify the time course of PEM-induced feedback upregulation of EGFR signaling, we monitored changes in EGFR signaling pathway activity over time in HCC827 cells exposed to 1 μM PEM. EGFR signaling underwent dramatic feedback upregulation starting at 72 h after PEM exposure (Figure 9G). Both pERK/ERK and pEGFR/EGFR ratios reached levels twofold higher than those at 0 h, while p-Akt signaling gradually returned to baseline (0 h) levels. These results demonstrate that during PEM-induced feedback upregulation of EGFR signaling, reactivation of the ERK1/2 pathway was more pronounced than that of the Akt pathway. Exposure to 50 nM OSI completely suppressed EGFR signaling within 12 h (Figure 9H). Based on our hypothesis illustrated in Figure 9I, when using the sequential PEM → OSI combination strategy, the subsequent administration of OSI effectively suppresses the feedback EGFR signaling induced by PEM. Conversely, when employing the sequential OSI → PEM strategy, the absence of sustained EGFR inhibition by OSI during PEM exposure may allow reactivation of EGFR signaling [19].

To verify whether inhibition of ERK1/2 signaling indeed synergizes with the late-phase effects of PEM, we evaluated the inhibition rate (Figure 9C), apoptosis rate (Figure 9J), and apoptotic signaling (Figure 9K,L) under the sequential combination of PEM and the ERK1/2 inhibitor GDC0994. The results demonstrated that GDC0994-mediated inhibition of ERK1/2 effectively enhanced PEM-induced apoptosis during its late-phase effects, and this pro-apoptotic enhancement was Bim-dependent (Figure 9L). These findings indicate that inhibiting PEM-induced feedback upregulation of EGFR signaling, particularly the downstream ERK1/2 signaling, strongly synergizes with PEM’s late-phase effects. Subsequently, we monitored the time course of EGFR and ERK1/2 signaling under PEM → OSI sequencing strategy to verify whether both EGFR and ERK signaling were inhibited by the subsequently administered EGFR-TKI as anticipated. As demonstrated in Figure 9M, subsequent administration of OSI effectively suppressed both PEM-induced EGFR signaling and ERK signaling.

We also compared the levels of EGFR signaling suppression achieved with different administration sequences at the endpoint in both PC9 and HCC827 cell lines (Figure 9N,O). Consistent with our hypothesis (Figure 9I) and prior reports on PEM–almonertinib combinations [18], the sequential PEM → OSI strategy resulted in the lowest p-EGFR/EGFR ratio in both cell lines and significantly reduced pERK/ERK levels compared to other sequential strategies in PC9 cells (Figure 9O).

Finally, the EC50 of OSI for pEGFR/EGFR inhibition was compared between (1) sequential PEM 48 h → OSI 24 h regimens and (2) 48h OSI monotherapy (Figure 9P). The EC50 values under these two conditions were 18.29 nM and 14.49 nM, respectively. These comparable values indicate that PEM neither enhances nor impairs OSI’s inhibitory efficacy against the EGFR receptor. This contrasts with reported findings where PEM potentiates almonertinib’s inhibition of EGFR [18]. It is noteworthy that in the PEM → OSI sequencing regimen, the EC50 of the EGFR inhibition curve for OSI remains unchanged, but the Hill slope decreases. This may result from compensatory EGFR signaling counteracting OSI (Figure 9P).

In summary, PEM-induced feedback EGFR signaling emerged at a very late stage of PEM exposure (Figure 9G). Consequently, the concurrent PEM + OSI combination strategy cannot achieve synergistic effects through early-phase EGFR suppression. Conversely, in the sequential PEM → OSI strategy employing a 48 h interval, OSI administration precisely coincides with the window of PEM-induced compensatory EGFR signaling while avoiding the undesirable effects associated with premature OSI delivery. This effectively suppresses compensatory EGFR signaling, thereby enabling synergistic efficacy.

### 3.9. Sequence-Dependent Synergistic Effects Between PEM and OSI in HCC827 Tumor-Bearing Balb/C Nude Mice

**Figure 10:**
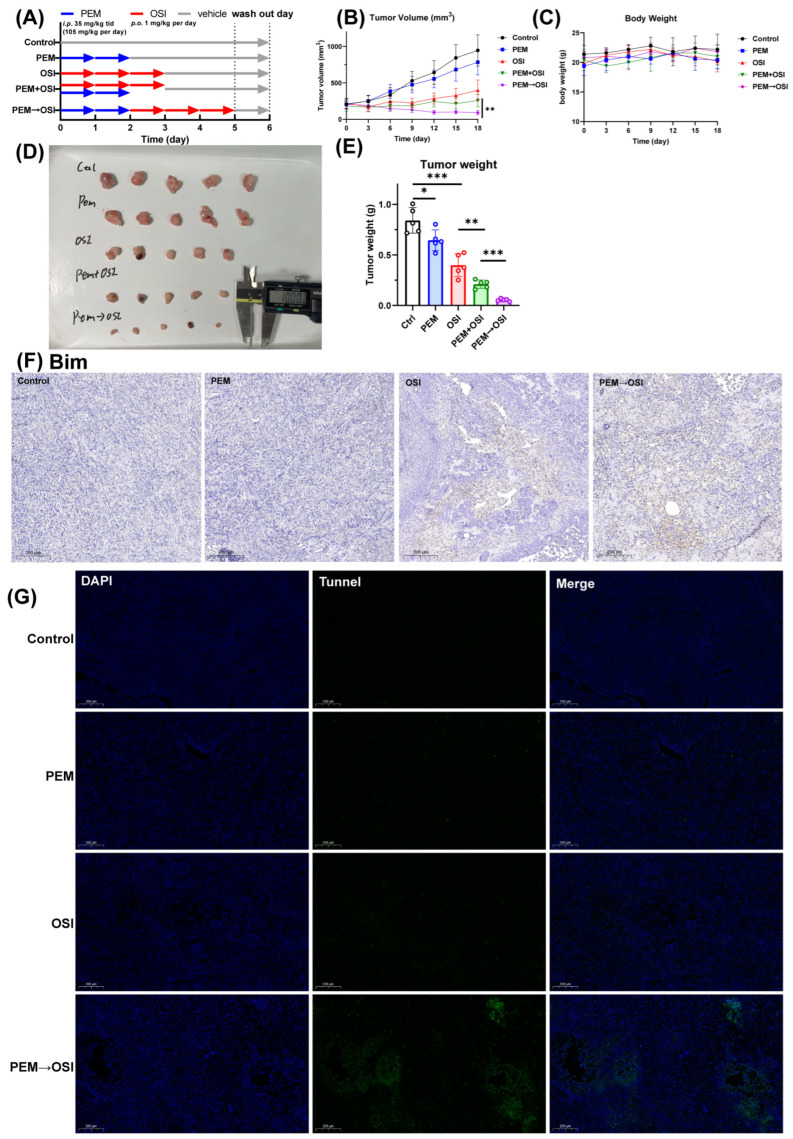
**Sequence-dependent synergistic effects between PEM and OSI in HCC827 tumor-bearing Balb/C nude mice**. (**A**) Schematic of the dosing regimen for each group per treatment cycle. Three treatment cycles were employed. (**B**,**C**) Tumor growth curves (**B**) and body weight changes (**C**) of HCC827 xenograft mice treated with PEM and OSI alone or in combination with different sequential strategies (*n* = 5). (**D**,**E**) HCC827 tumor-bearing mice were sacrificed post three-cycle drug administration, and the tumors were dissected, photographed (**D**), and weighed (**E**) (*n* = 5). (**F**) Representative immunohistochemical staining of Bim in tumor sections for each group (*n* = 3). (**G**) Representative TUNEL staining in tumor sections for each group (*n* = 3) (scale bar 200 µm). All data were presented as mean ± SD, * *p* < 0.05, ** *p* < 0.01, *** *p* < 0.001.

Based on in vitro experimental data (Figure 2) and our hypothesized mechanism (Figure 6, Figure 7, Figure 8 and Figure 9), we further validated the reliability of these findings in HCC827 Balb/c nude mice xenograft model. As illustrated in Figure 10A (shows a diagram of one treatment round; three rounds were performed in total), we compared the tumor-inhibitory efficacy of concurrent PEM and OSI administration versus the sequential PEM → OSI strategy under conditions of equivalent total drug exposure while excluding the sequential OSI → PEM regimen due to its obvious antagonistic effects and lack of clinical relevance. To prevent the OSI-mediated G1-phase arrest from antagonizing the subsequent PEM therapy in the PEM → OSI strategy, a 1 d washout period was implemented. Given that PEM is eliminated extremely rapidly in mice (*t*_1/2_ = 35 min; in-house data), we administered PEM three times daily at 35 mg/kg per dose with 4 h intervals to simulate human PEM exposure. The total daily PEM exposure (105 mg/kg/day) was consistent with the previously used PEM dosage (100 mg/kg/day) in prior studies [4,16,18,19].

The results of the in vivo study are presented in Figure 10. The concurrent PEM + OSI strategy exhibited only mild synergy, whereas the sequential PEM → OSI strategy demonstrated strong synergy (Figure 10B,D,E). At the endpoint, the mean tumor weights relative to the OSI group were 52.87% (PEM + OSI) and 13.50% (PEM → OSI), respectively. Significant differences were observed in tumor weight for both the PEM + OSI versus OSI and the PEM + OSI versus PEM → OSI comparisons (Figure 10E). TUNEL staining of tumor sections showed that the PEM → OSI group exhibited the strongest TUNEL fluorescence signal (Figure 10G). Immunohistochemistry for Bim demonstrated high Bim protein expression in both the OSI group and the PEM → OSI group (Figure 10F). These pathological section results, consistent with our in vitro findings (Figure 7 and Figure 8) and other in vitro reports [4,16,19], further confirmed in vivo that OSI strongly enhances PEM-induced apoptosis by upregulating the pro-apoptotic protein Bim.

Throughout the entire three-week drug treatment period, food and water intake showed no significant changes in any group of mice, and body weight remained stable (Figure 10C). This indicates that neither the concurrent PEM + OSI strategy nor the sequential PEM → OSI strategy raised safety concerns. This was further supported by HE-stained liver and kidney tissue sections.

## 4. Discussion

A major research focus in combining chemotherapy with molecularly targeted therapies is enhancing efficacy by optimizing the administration sequence [38,55,56]. Given the demonstrated superiority of the sequential PEM → almonertinib combination over its concurrent administration [18,19], the sequential PEM → OSI approach is anticipated to be more promising than the concurrent administration regimen. Among the three combination strategies (concurrent PEM + OSI, sequential PEM → OSI, and sequential OSI → PEM), the sequential OSI → PEM strategy was not considered due to its evident cell cycle-based antagonistic effects (Figure 4I) [18,19]. However, the relative merits of concurrent PEM + OSI versus sequential PEM → OSI administration remain to be evaluated. Concurrent administration is widely adopted due to its better compliance, simpler dosing regimen, and avoidance of drug holiday [8,9]. However, the advantages of sequential administration have been demonstrated in both preclinical and clinical studies [18,19]. The core rationale of the sequential PEM → OSI strategy lies in temporally separating PEM-induced early S-phase arrest (necessary for its late cytotoxicity) from OSI-mediated G1-phase blockade to avoid early interference while preserving the overlap between PEM-induced late-phase apoptosis and OSI treatment, thereby exploiting OSI’s synergistic pro-apoptotic effects. The optimal sequential schedule must allow PEM to induce potent late-phase cytotoxicity (Figure 1B) while enabling timely synergy with OSI’s pro-apoptotic effects. To determine this schedule, this study systematically investigates the mechanism of the sequential strategy from a pharmacodynamic perspective, characterizing key post-PEM events, including S-phase arrest, DNA damage, apoptotic signaling, and EGFR signaling pathways (Figure 4, Figure 5, Figure 6, Figure 7, Figure 8 and Figure 9). We employ time-course profiles of key pharmacodynamic biomarkers to visualize the pharmacodynamic interactions between PEM and OSI at different sequential schedules. Finally, we preliminarily validated our conclusions in HCC827 xenograft mice.

We divided the pharmacodynamic process post-PEM treatment into early and late phases. The early phase is characterized by nucleotide depletion, S-phase arrest (Figure 4I), replication fork stalling, and DNA damage accumulation (Figure 6A), where effect accumulation directly determines late-phase response intensity. The late phase features DNA damage peaking (Figure 6A), apoptotic signal activation (Figure 7A), and feedback EGFR signaling activation (Figure 9G). OSI antagonizes PEM’s early effects via G1-phase blockade, which counteracts S-phase arrest and DNA damage accumulation (Figure 4H,I and Figure 6L,M). Conversely, OSI enhances PEM’s late-phase cytotoxicity by (a) suppressing Rad51/TS (Figure 6J,K,N), (b) upregulating Bim (Figure 8A), and (c) inhibiting feedback EGFR signaling (Figure 9M). All PEM late-phase effects potentiated by OSI occurred after 48 h of PEM exposure. Therefore, the 48 h interval sequential PEM → OSI strategy allows for sufficient accumulation of PEM’s early effects while timely engaging OSI’s pro-apoptotic action, thus maximizing synergy. ERK1/2 was identified as the critical mediator of synergy between PEM and EGFR-TKI in this study, with its inhibition leading to Rad51 downregulation and Bim upregulation (Figure 9D,E). Similar to PEM, the combination of 5-FU and the MEK inhibitor selumetinib also exhibits sequence-dependent synergy, with administration of 5-FU followed by selumetinib showing the strongest effect [55]. The HDAC inhibitor ITF2357 synergizes with PEM, in part through mechanisms, including downregulation of Rad51 and TS [43,45]. Notably, the synergy between ITF2357 and PEM is also sequence-dependent, with the administration sequence of PEM followed by ITF2357 yielding the optimal effect. This demonstrates that downregulation of Rad51 and TS potentiates the late-stage effects of PEM [45]. The cell signaling-time curves for the sequential PEM → OSI strategy demonstrate that after 48 h of PEM exposure, subsequent addition of OSI leads to a sharp intensification of apoptotic signaling, Bim, and DNA damage signaling (Figure 6J and Figure 8A). Furthermore, the sequential combination prevented the 72 h feedback upregulation of EGFR signaling observed with PEM monotherapy (Figure 9M,O). The 48 h interval sequential PEM → OSI strategy also demonstrated the strongest synergistic effect in HCC827 xenograft mouse (Figure 10B,D,E), while the concurrent PEM + OSI strategy only exhibited mild synergy. Tumor section analysis through TUNEL staining and Bim immunohistochemistry further confirmed that the sequential PEM → OSI regimen significantly enhances PEM-induced apoptosis effects by upregulating the pro-apoptotic protein Bim (Figure 10F,G).

This study is the first to identify the sequential interval as a critical determinant of synergistic efficacy in the PEM → EGFR-TKI sequential strategy. A 24 h interval generated optimal synergy only in PEM-sensitive PC9 cells (Figure 2C), while a 48 h interval demonstrated optimal synergy across OSI-sensitive cell lines (Figure 2D,F,H). As illustrated in Figure 7J, an in-depth analysis of the mechanism of OSI synergizing with PEM reveals that OSI exerts synergistic effects by promoting apoptosis through downregulating Rad51 and TS and upregulating the pro-apoptotic protein Bim rather than directly inducing apoptosis. This pro-apoptotic effect requires cells to be in a specific “damaged” state. This synergy is preconditioned on PEM having already induced sufficient cellular damage. However, in PEM-moderately sensitive cells such as HCC827, exposure to PEM for 24 h manifests as weak apoptotic signaling (Figure 7A), low levels of DNA damage (Figure 6A), and moderate S-phase arrest (Figure 4I). This indicates that the 24 h exposure is insufficient to induce the required “damaged” state. A significant difference in late-stage inhibition rates was observed between cells exposed to PEM for 24 h versus 48 h (Figure 1B) in PEM-moderate NSCLC cells. Concurrently, dramatic dynamic changes in DNA damage markers (Figure 6A) and apoptotic signaling occurred between 24 and 48 h (Figure 7A), further confirming that by 48 h, cells had entered a definitive “damaged” state, whereas the damage state at 24 h remained extremely faint. Consequently, sequential dosing with a 48 h interval generates strong synergistic effects, whereas no detectable synergy occurs with a 24 h interval PEM → OSI regimens (Figure 7J). Notably, although PC9 cells already demonstrated synergistic effects with the 24 h interval sequential PEM → OSI strategy, the 48 h interval further enhanced synergistic potency (Figure 2C,D and Tables S4 and S5; the Bliss score increased from −0.022 at the 24 h sequential interval scenario to 0.053 at the 48 h sequential interval scenario).

PEM and OSI concentrations used in this study were all within clinically translatable drug exposure levels, and consideration for their experimental concentration design was presented in Supplementary Material Section S4 [31,33,34,57]. Clinical-free concentration of OSI under steady state fluctuated between 10 and 50 nM (Supplementary Material Section S4). Concentration-response analysis showed PEM → OSI regimens did not alter OSI’s EC50 for Bim activation or p-EGFR inhibition (Figure 8D and Figure 9P), indicating unaffected target binding/Bim upregulation. However, EC50s for γ-H2AX induction (DNA damage, Figure 6N) and apoptosis protein activation (Figure 8E–G) were significantly lower, indicating heightened OSI sensitivity in PEM-treated cells. Nonlinear fitting showed that PEM → OSI regimens increased DNA damage 2.7-fold and apoptotic signaling 3.5–5.5-fold versus PEM alone (Figure 6N and Figure 8E–G). This enhancement likely stems from OSI’s potent pro-apoptotic action on cells in a PEM-induced “damage-heightened” state. This relatively low EC50 value for PEM → OSI regimens indicates that, despite dynamic changes in OSI-free concentration, tumor cells at PEM induced late-phase inhibition state subsequently treated with relatively low free concentrations of OSI can still induce significant DNA damage and apoptotic signals. The dynamic concentrations of OSI are sufficient to continuously elicit potent DNA damage and apoptotic signals. In contrast, OSI monotherapy may only effectively induce DNA damage and apoptosis signals during limited time windows at relatively high free OSI concentration.

Whether the sequential PEM → EGFR TKI combination benefits only EGFR-mutant [18,58] or extends to wild-type contexts [19,21,22] remains unresolved in prior studies. Given that OSI treatment is clinically restricted to EGFR-mutant-positive NSCLC patients, our investigation utilized solely EGFR-mutant cell models. Importantly, absence of synergy in NCI-H1650 cells (PTEN loss, constitutive ERK/Akt activation) confirms that the sequential synergy requires preserved sensitivity to EGFR-TKIs (Figure S1 and Figure 2I,J). Conversely, PC9 and HCC827 cells exhibiting high EGFR and Bim expression (Figure S1) show superior synergy with sequential PEM → OSI treatment (Figure 2D,F and Tables S5 and S7), outperforming the higher-exposure PEM + OSI-2 concurrent strategy.

The sequential OSI → PEM strategy significantly impairs PEM-induced S-phase arrest and DNA damage accumulation (Figure 4I and Figure 6L). This indicates that during multi-cycle therapy using the PEM → OSI sequence, residual G1 arrest from prior treatment cycles severely compromises subsequent PEM cytotoxicity (Figure 4I). Critically, the synergy between OSI and PEM depends on intact late-stage cytotoxicity of PEM. To mitigate interference, transient withholding of OSI post-sequential therapy enables dissipation of G1 arrest prior to subsequent PEM administration [19]. However, as an irreversible EGFR inhibitor, OSI requires receptor resynthesis to re-establish EGFR signaling [39]. This necessity may entail prolonged discontinuation periods, raising concerns that disease progression risks during extended drug holidays could outweigh the benefits of sequential therapy (Figure S4). Therefore, we further determined the washout period for OSI-induced G1 phase arrest (Figure 5). Based on our results, the G1-arrest reversed within 48 h after OSI removal, even in OSI-sensitive HCC827 and PC9 cells (Figure 5). This suggests that the sequential PEM → OSI combination does not require a long drug holiday between treatments, strengthening our confidence in the clinical implementation of the sequential PEM → OSI regimens.

When PEM and OSI are administered simultaneously, OSI does not immediately block all cells in G1 phase, with a substantial proportion of cells persisting in S-phase. By 48 h, approximately 30% of cells remained arrested in S-phase by PEM (Figure 4H). This implies that during synchronous PEM + OSI co-treatment, PEM still induces partial S-phase arrest, generating a later-phase effect that OSI can synergize with, although this effect is substantially weaker than that in sequential PEM → OSI strategy. Clinically, concurrent PEM + OSI administration requires no sequential interval adjustments or treatment breaks between cycles, achieving maximum OSI exposure duration. In 21 d PEM cycles, the sequential PEM → OSI regimen (with 2 d dosing interval and 2 d inter-cycle break) reduces OSI exposure by 4 d per cycle compared to concurrent PEM + OSI therapy (reducing about 16.7% total OSI exposure). Thus, clinical translation of sequential combination therapy requires careful consideration of the trade-off between synergistic benefits from the sequential PEM → OSI strategy and reduced OSI exposure. Nevertheless, the stronger synergistic potency of PEM → OSI sequencing observed in PC9 and HCC827 cells, despite its lower total drug exposure compared to the PEM + OSI-2 concurrent strategy significantly reinforces confidence in this sequential combinatorial approach (Figure 2A,B,D,F and Tables S5 and S7).

This study provides critical insights for the clinical combination of PEM → OSI regimens: (1) the 48 h interval sequential PEM → OSI strategy demonstrated optimal synergy in both PEM-sensitive and moderate-sensitive patients (Figure 2D,F,H and Tables S5, S7 and S9); (2) synergy appeared OSI-response dependent, with NSCLC cell lines exhibiting high EGFR/Bim expression likely benefiting maximally from this regimens; and (3) to avoid interfering with the next PEM cycle, brief interruption of OSI administration may be necessary during sequential PEM → OSI therapy across multiple cycles. The treatment pause may not exceed 48 h (Figure 5).

However, this study also has several limitations: (1) The roles of other DNA damage repair-associated proteins (BRCA1 and DNA-PKcs) [59], EGFR-TKI-related pro-apoptotic regulators (e.g., PUMA) [26], and EGFR nuclear translocation [60], as well as p53 genetic variations (where p53 protein serves as a critical mediator linking DNA damage and apoptotic signaling) in sequential PEM → OSI synergistic effects, remain incompletely explored. (2) Although upregulation of reactive oxygen species (ROS) levels is a key pharmacological effect of PEM [36] (besides DNA damage), as documented in the literature, the interplay between this ROS induction and the pharmacological effects of OSI remains unexplored in this study. (3) The interplay between PI3K/Akt pathway inhibition and PEM’s late-phase effects remains unexplored. (4) The impact of sequential strategies on tumor migration and invasion, as well as clonogenic potential, has not been assessed [18,19]. (5) The potential of sequential PEM → OSI administration to delay drug resistance remains uninvestigated [4]. (6) While FLAURA2 data support the safety of concurrent PEM + OSI therapy (overlapping drug exposure) [61,62] and staggered sequencing exposure strategy may mitigate toxicity risks versus concurrent exposure, systematic toxicity profiling of sequential PEM → OSI regimens in proliferating normal cells in lung tumor-originating tissues (e.g., bronchial epithelial cells) remains crucial and should be explored in the future.

## 5. Conclusions

In conclusion, the 48 h interval PEM → OSI strategy showed superior synergy efficacy in vitro and in vivo. PEM exposure for 48 h enables completion of S-phase arrest/DNA damage accumulation and apoptotic priming. OSI then potentiates these late effects by inhibiting Rad51/TS, upregulating Bim, and suppressing feedback EGFR signaling, significantly enhancing PEM-induced apoptosis.

## Data Availability

The data that support the findings of this study are available from the corresponding author upon reasonable request.

## Supplementary Materials

The following supporting information can be downloaded at: https://www.mdpi.com/article/10.3390/pharmaceutics17081044/s1, Supplementary Materials including: Section S1: Supplementary Tables; Section S2: Supplementary Figures; Section S3: Corresponding Western Blot image for Figure 6, Figure 7, Figure 8 and Figure 9; Section S4: Considerations in concentration selection for in vitro assays; Section S5: STR certifications for NSCLC cell lines used in this study.
