# Supplementary material for: Pharmacodynamic-Driven Sequence-Dependent Synergy Effects in Pemetrexed-Osimertinib Combination Against Non-Small Cell Lung Cancer (NSCLC): Optimizing Synergy Through Sequential Interval
Source: Pharmaceutics. 2025 Aug 12;17(8):1044. doi: 10.3390/pharmaceutics17081044 (PMC12389513; doi:10.3390/pharmaceutics17081044)
Supplement: Supplementary file 1 [file pharmaceutics-17-01044-s001.zip › pharmaceutics-3727392-supplementary.pdf]

Supporting Information for Original article:

Pharmacodynamic-Driven Sequence-dependent Synergy Effects in Pemetrexed-Osimertinib Combination against Non-Small Cell Lung Cancer (NSCLC): Optimizing Synergy through sequential interval

This file includes:

1. **Section S1: Supplementary Tables**
2. **Section S2: Supplementary Figures**
3. **Section S3: Corresponding Western Blot image for figure 6, 7, 8, 9**
4. **Section S4: Considerations in Concentration Selection for *In Vitro* Assays**
5. **Section S5: STR Certifications for NSCLC cell lines used in this study**
6. **Reference for Supplementary materials**

1. Section S1: Supplementary Tables

Table S1. List of chemicals, reagents and cell lines.

Chemicals and reagents	Catalog	Sources
Pemetrexed Disodium Salt Heptahydrate	P303530	Aladdin (Shanghai, China)
Osimertinib Mesylate	BD295391	Bidepharm (Shanghai, China)
GDC0994	HY-15947	MedChemExpress (Shanghai, China)
MK2206	M671171	Aladdin (Shanghai, China)
ABT263	A276560	Aladdin (Shanghai, China)
BIM BH3	HY-P1527	MedChemExpress (Shanghai, China)
Protein Transfection Reagent	HY-K2016	MedChemExpress (Shanghai, China)
RNAimax	13778150	Invitrogen (Carlsbad, CA, USA)
EDU assay kit 594	C0078L	Beyotime (Shanghai, China)
Cell Cycle Kit		Fcmacs Biotechnology (Nanjing, China)
Halt protease and phosphatase inhibitor cocktail (100×)	78442	Invitrogen (Carlsbad, CA, USA)
5× SDS-PAGE loading buffer	WB2001	NCM-bio (Suzhou, China)
MTT	ST1537-5g	Beyotime (Shanghai, China)
PC-9	SCSP-5085	Cell Bank of the Chinese Academy of Sciences (Shanghai, China)
NCI-H1650	SCSP-592	Cell Bank of the Chinese Academy of Sciences (Shanghai, China)
HCC-827	SCSP-538	Cell Bank of the Chinese Academy of Sciences (Shanghai, China)
NCI-H1975	SCSP-597	Cell Bank of the Chinese Academy of Sciences (Shanghai, China)
Enhanced Chemiluminescence		NCM-bio (Suzhou, China)
Accutase	00-4555-56	Invitrogen (Carlsbad, CA, USA)
Caspase 3/7 Activity kit	C1077M	Beyotime (Shanghai, China)
Annexin V-FITC/PI Apoptosis Kit	AT101	MULTISCIENCES (Hangzhou, China)
Restore PLUS Western Blot Stripping Buffer	46430	Invitrogen (Carlsbad, CA, USA)
Matrix-Gel (High Concentration)	C0383-5ml	Beyotime (Shanghai, China)

Table S2. List of antibodies used in the study.

Protein target	Phosphorylation site/ cleavage site	Catalog Number	Manufacturer	Application
EGFR		4267S	Cell Signaling Technology (Danvers, MA, USA)	Western blot
p-EGFR	Y1068	3777S	Cell Signaling Technology (Danvers, MA, USA)	Western blot
Akt		4691S	Cell Signaling Technology (Danvers, MA,	Western blot

			USA)	
p-Akt	S473	4060s	Cell Signaling Technology (Danvers, MA, USA)	Western blot
ERK		4695s	Cell Signaling Technology (Danvers, MA, USA)	Western blot
p-ERK	T202/Y204	4370S	Cell Signaling Technology (Danvers, MA, USA)	Western blot
GAPDH			Cell Signaling Technology (Danvers, MA, USA)	Western blot
β-tubulin		A12289	ABclonal (Wuhan, Hubei, China)	Western blot
β-Actin		20536-1-AP	Proteintech (Rosemont, IL, USA)	Western blot
RAD51		8875S	Cell Signaling Technology (Danvers, MA, USA)	Western blot
TS		9045S	Cell Signaling Technology (Danvers, MA, USA)	Western blot
P53		A19585	ABclonal (Wuhan, Hubei, China)	Western blot
p-P53	S15	AP1504	ABclonal (Wuhan, Hubei, China)	Western blot
γ-H2AX	S139	9718S	Cell Signaling Technology (Danvers, MA, USA)	Western blot
Caspase 3		14220S	Cell Signaling Technology (Danvers, MA, USA)	Western blot
Cl-Caspase 3	D175	9664S	Cell Signaling Technology (Danvers, MA, USA)	Western blot

Caspase 7		12827S	Cell Signaling Technology (Danvers, MA, USA)	Western blot
Cl-Caspase 7	Asp198	8438S	Cell Signaling Technology (Danvers, MA, USA)	Western blot
Caspase 9		9502S	Cell Signaling Technology (Danvers, MA, USA)	Western blot
Cl-Caspase 9	Asp315	20750S	Cell Signaling Technology (Danvers, MA, USA)	Western blot
PARP		9532S	Cell Signaling Technology (Danvers, MA, USA)	Western blot
Cl-PARP	Asp214	5625S	Cell Signaling Technology (Danvers, MA, USA)	Western blot
Bim		2933S	Cell Signaling Technology (Danvers, MA, USA)	Western blot
Ki67		MA5-14520	Invitrogen (Carlsbad, CA, USA)	IF and FC
CDK4		11026-1-AP	Proteintech (Rosemont, IL, USA)	Western blot
CDK6		14052-1-AP	Proteintech (Rosemont, IL, USA)	Western blot
CDK2		18048T	Cell Signaling Technology (Danvers, MA, USA)	Western blot
P27		3688S	Cell Signaling Technology (Danvers, MA, USA)	Western blot
SKP2		2652T	Cell Signaling Technology	Western blot

		(Danvers, MA, USA)	
Cyclin A2	67955S	Cell Signaling Technology (Danvers, MA, USA)	Western blot
Cyclin E1	20808S	Cell Signaling Technology (Danvers, MA, USA)	Western blot
Cyclin D1	55506S	Cell Signaling Technology (Danvers, MA, USA)	Western blot

Table S3. Genetic Backgrounds of the Four EGFR-Mutant NSCLC Cell Lines

Title 1	PC9	HCC827	NCI-H1975	NCI-H1650
EGFR mutation	19 del [1] [1]	19 del [1]	L858R [1] [2]	19 del [2]
EGFR expression	High [3]	High [3] [3]	Low [3] [3]	Low [3] [3]
EGFR related resistance mutation	NA[4]	NA	T790M [1]	NA
EGFR sub path way related resistance mutation	NA	NA	NA	PTEN loss
Bim expression	high [3]	high [3]	low [3]	low [3]
Bim related mutation	NA	NA	NA	NA
p53 related mutation	p53-R248Q [4,5] [5]	p53-p.V218del [4,5] [6]	p53-R273H [4,5] [7]	p53-p.V225fs [6] [8]
Rad51 related mutation	NA	NA	NA	NA
Rad51 expression	-	Relatively low[9]	-	-
TS related mutation	The TS gene exhibits polymorphisms, but no disease-causing mutations were identified/found.			
TS expression	-	Relatively low[9]	-	-

[1] 19del: EGFR exon 19 deletions, EGFR c.2235_2249del15 (p.Glu746_Ala750del)
[2] L858R: EGFR c.2573T>G (p.Leu858Arg), rs121434568
[3] Expression levels were inferred from the results in Figure S2.
[4] NA (not applicable): No relevant reports found
[5] p53-R248Q: TP53 c.743G>A (p.R248Q), rs11540652,
[6] p53-p.V218del: TP53 c.652_654delTGT (p.Val218del)

Table S4 Summary of Bliss score for PC9 with a 24 h sequential interval under different sequencing strategies

Conc. PEM (nM)	Conc. OSI (nM)	PEM	OSI	PEM→OSI		OSI→PEM		PEM+OSI	
		**inhibition%**	**inhibition%**	**inhibition%**	**Bliss**	**inhibition%**	**Bliss**	**inhibition%**	**Bliss**
52.5	11.25	30.00	12.12	43.89	0.054	9.77	-0.287	38.99	0.005
70	15	39.09	28.52	57.09	0.006	35.48	-0.210	46.94	-0.095
105	22.5	47.68	48.13	69.04	-0.038	53.46	-0.194	62.39	-0.105
140	30	55.71	56.91	74.86	-0.061	62.13	-0.188	68.27	-0.126
210	45	60.22	67.06	79.65	-0.072	70.57	-0.163	71.94	-0.150
Mean				-0.022		-0.208		-0.094	

The calculation method for Bliss Score was as follows:

$Bliss\ Scroe = E_{AB} - (E_A + E_B - E_A \cdot E_B)$. E represents the inhibition rate normalized to a range of 0 to 1. Where E_{AB} represents the observed inhibition rate during combination therapy, whereas E_A and E_B represent the inhibition rate during monotherapy.

Table S5 Summary of Bliss score for PC9 with a 48 h sequential interval under different sequencing strategies

Conc. PEM (nM)	Conc. OSI (nM)	PEM	OSI	PEM→OSI		OSI→PEM		PEM+OSI	
		**inhibition%**	**inhibition%**	**inhibition%**	**Bliss**	**inhibition%**	**Bliss**	**inhibition%**	**Bliss**
52.5	11.25	30.51	15.30	46.88	0.057	19.18	-0.220	20.05	-0.211
70	15	40.96	23.00	61.32	0.068	23.47	-0.311	28.66	-0.259
105	22.5	50.31	31.73	74.83	0.088	37.08	-0.290	44.27	-0.218
140	30	57.83	40.17	81.12	0.064	52.23	-0.225	53.61	-0.212
210	45	69.45	54.73	84.86	-0.013	62.77	-0.234	58.67	-0.275
Mean				0.053		-0.256		-0.235	

Table S6 Summary of Bliss score for HCC827 with a 24 h sequential interval under different sequencing strategies

Conc. PEM (nM)	Conc. OSI (nM)	PEM	OSI	PEM→OSI		OSI→PEM		PEM+OSI	
		**inhibition%**	**inhibition%**	**inhibition%**	**Bliss**	**inhibition%**	**Bliss**	**inhibition%**	**Bliss**
300	6.5	17.11	21.84	25.25	-0.100	9.06	-0.262	37.67	0.025
450	9.75	22.81	41.82	40.23	-0.149	31.17	-0.239	44.09	-0.110
600	13	23.13	46.41	40.21	-0.186	36.96	-0.218	45.84	-0.130
900	19.5	41.75	60.59	56.38	-0.207	58.30	-0.187	57.72	-0.193
Mean					-0.180		-0.215		-0.144

Table S7 Summary of Bliss score for HCC827 with a 48 h sequential interval under different sequencing strategies

Conc. PEM (nM)	Conc. OSI (nM)	PEM	OSI	PEM→OSI		OSI→PEM		PEM+OSI	
		inhibition%	inhibition%	inhibition%	BLISS	inhibition%	BLISS	inhibition%	BLISS
100	2.17	7.08	0.13	11.89	0.047	7.67	0.005	6.68	-0.005
150	3.25	16.67	0.92	20.68	0.032	14.83	-0.026	4.21	-0.132
225	4.88	22.82	15.70	30.96	-0.040	41.52	0.066	21.52	-0.134
300	6.50	32.06	30.86	43.65	-0.094	37.87	-0.152	35.42	-0.176
450	9.75	39.22	33.30	52.08	-0.074	30.03	-0.294	43.68	-0.158
600	13.00	41.44	37.37	54.72	-0.086	34.79	-0.285	43.55	-0.198
900	19.50	42.20	49.00	64.47	-0.060	49.11	-0.214	52.66	-0.179
Mean					-0.039		-0.129		-0.140

Table S8 Summary of Bliss score for NCI-H1975 with a 24 h sequential interval under different sequencing strategies

Conc. PEM (nM)	Conc. OSI (nM)	PEM	OSI	PEM-OSI		OSI-PEM		PEM+OSI	
		inhibition%	inhibition%	inhibition%	BLISS	inhibition%	BLISS	inhibition%	BLISS
210	24	24.44	51.82	52.88	-0.107	50.39	-0.132	55.88	-0.077
280	32	33.02	56.34	57.69	-0.131	56.87	-0.139	61.51	-0.092
420	48	31.43	59.65	58.99	-0.133	60.01	-0.123	62.33	-0.100
Mean					-0.124		-0.131		-0.090

Table S9 Summary of Bliss score for NCI-H1975 with a 48 h sequential interval under different sequencing strategies

Conc. PEM (nM)	Conc. OSI (nM)	PEM	OSI	PEM-OSI		OSI-PEM		PEM+OSI	
		inhibition%	inhibition%	inhibition%	BLISS	inhibition%	BLISS	inhibition%	BLISS
46.662	5.3328	6.72	17.72	14.07	-0.092	1.97	-0.213	9.49	-0.138
70	8	14.51	1.55	29.09	0.132	19.76	0.039	6.51	-0.093
105	12	27.59	28.92	39.81	-0.087	29.84	-0.187	27.29	-0.212
140	16	34.33	31.94	47.48	-0.078	33.30	-0.220	41.13	-0.142
210	24	50.07	44.12	56.86	-0.152	44.99	-0.271	53.31	-0.188
280	32	55.08	51.76	64.89	-0.134	48.32	-0.300	57.80	-0.205
420	48	55.47	59.44	67.74	-0.142	56.01	-0.259	58.97	-0.230
Mean					-0.079		-0.202		-0.173

Table S10 Summary of Bliss score for NCI-H1650 with a 24 h sequential interval under different sequencing strategies

Conc. PEM (nM)	Conc. OSI (μM)	**PEM** inhibition%	**OSI** inhibition%	**PEM→OSI** inhibition%	BLISS	**OSI→PEM** inhibition%	BLISS	**PEM+OSI** inhibition%	BLISS
46.662	1.3332	2.05	19.23	18.47	-0.024	24.50	0.036	30.14	0.093
70	2	-0.29	17.08	18.58		21.26		24.74	
105	3	2.77	24.38	20.63	-0.058	25.20	-0.013	31.24	0.048
140	4	12.62	25.92	33.44	-0.018	30.25	-0.050	51.77	0.165
210	6	20.42	29.75	26.31	-0.178	37.64	-0.065	35.70	-0.084
280	8	18.80	46.39	38.06	-0.184	48.22	-0.082	80.21	0.237
420	12	27.48	98.49	83.34	-0.156	98.72	-0.002	98.75	-0.002
Mean				-0.119		-0.042		0.073	

Table S11 Summary of Bliss score for NCI-H1650 with a 48 h sequential interval under different sequencing strategies

Conc. PEM (nM)	Conc. OSI (μM)	**PEM** inhibition%	**OSI** inhibition%	**PEM→OSI** inhibition%	BLISS	**OSI→PEM** inhibition%	BLISS	**PEM+OSI** inhibition%	BLISS
46.662	1.3332	15.14	18.41	20.09	-0.10674	27.09	-0.03678	32.59	0.01822
70	2	19.28	16.64	21.74	-0.1097	29.28	-0.03435	39.25	0.065421
105	3	19.57	18.87	24.88	-0.09864	30.91	-0.03827	46.68	0.119409
140	4	32.42	26.63	41.23	-0.09192	37.65	-0.1277	52.25	0.01834
210	6	27.94	19.30	33.53	-0.08318	36.30	-0.05549	50.05	0.082053
280	8	37.24	42.64	56.73	-0.07272	54.93	-0.09076	68.66	0.046577
420	12	39.62	98.90	81.96	-0.17378	98.95	-0.00389	99.19	-0.00151
Mean				-0.105240306		-0.055320306		0.049786837	

Table S12 siRNA Sequence for TS and Rad51

siRNA	Sequence (Sense strand)
siRad51	GGUGGUAGCUCAAGUGGAU tt
siTS	CUUUGGGAGAUGCACAUAUUUTT
siNC	UUCUCCGAACGUGUCACGU TT

Table S13 Silencing efficiency of the siRad51 and siTS knockdowns

	#1	#2	#3
siRad51	60.24%	96.50%	97.26%
siTS	50.67%	41.36%	77.68%

Silencing Efficacy% =$1 - \frac{Silencing}{control} \times 100\%$

SiRad51: #3 were final used in this study

SiTS: #3 were final used in this study

For siRad51

#1: GAGAUCAUACAGAUUACUA tt

#2: GUAGAGAAGUGGAGCGUAA tt

#3: GGUGGUAGCUCAAGUGGAU tt

For siTS

#1: GAGUGAUUGACACCAUCAATT

#2: GGGAGUUGACCAACUGCAATT

#3: CUUUGGGAGAUGCACAUAUUUTT

2. Section S2: Supplementary Figures

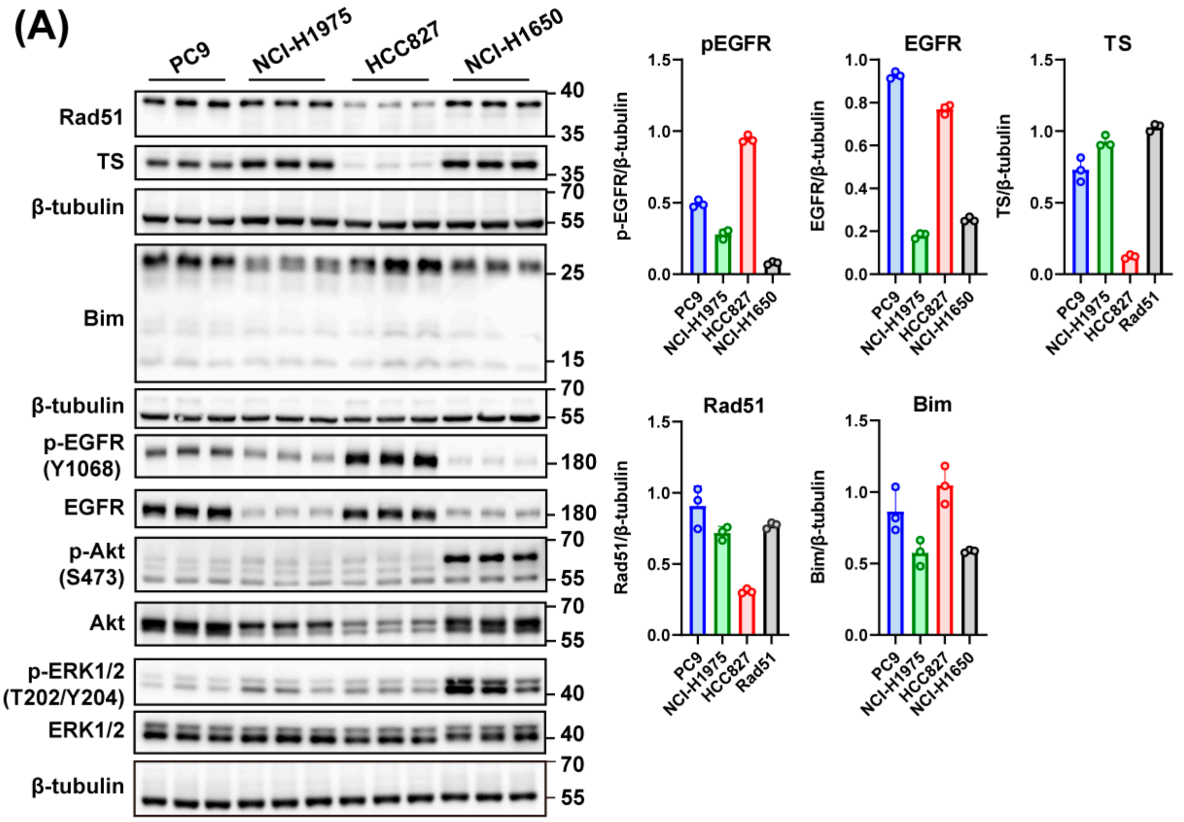

Figure S1 The expression levels of PEM and OSI Pharmacodynamic associated proteins in 4 NSCLC cell lines (*n* = 4).

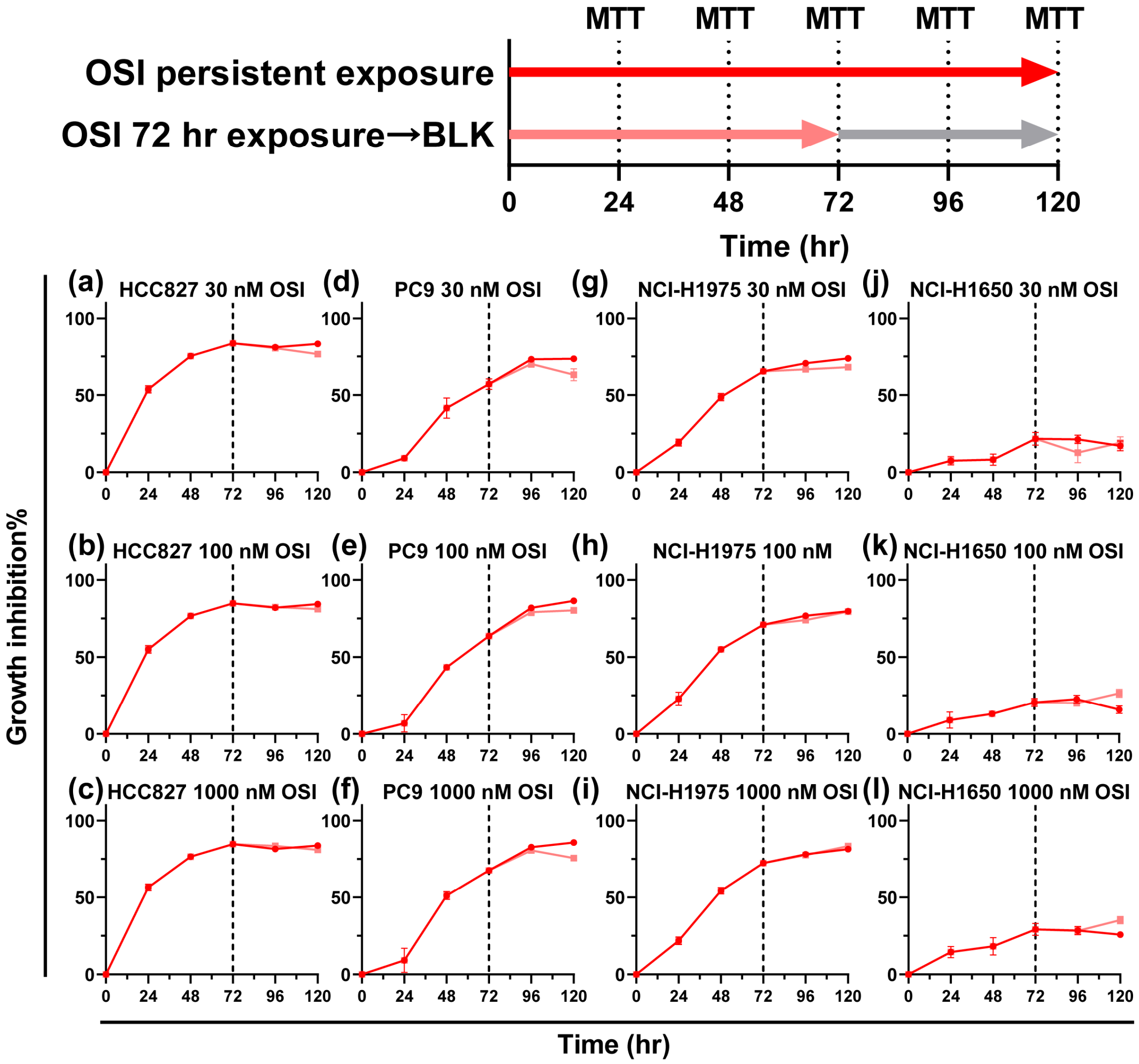

Figure S2 OSI-mediated growth inhibition plateaued after 72 h of OSI exposure. This figure illustrates the inhibition-time profiles of four EGFR-mutant-positive cell lines treated with varying OSI concentrations (30, 100, and 1000 nM) (n = 6).

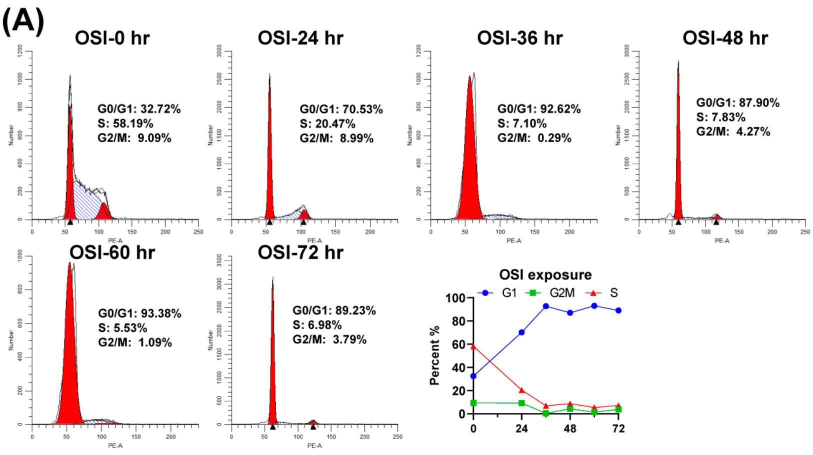

Figure S3 The time course of cell cycle distribution in HCC827 following 50 nM OSI treated for 72 h ($n = 3$).

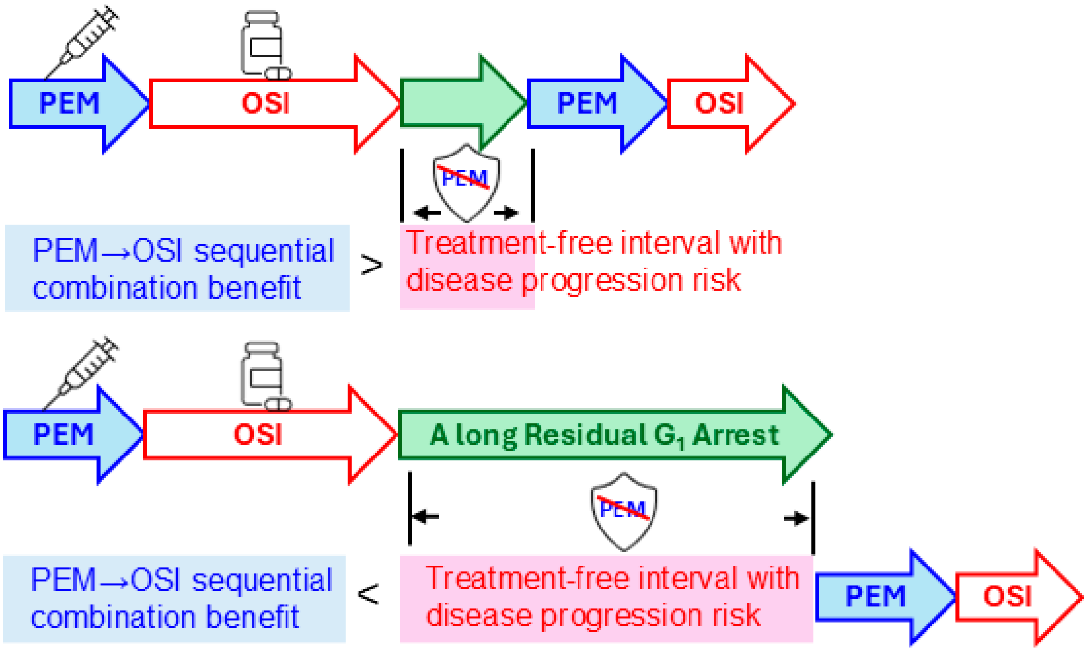

Figure S4 schematic illustration: A short G1 arrest decay duration enables PEM-OSI sequential therapy benefits to outweigh transient OSI withdrawal risks, whereas prolonged decay renders therapy benefits insufficient to offset long-term OSI discontinuation-driven progression risks.

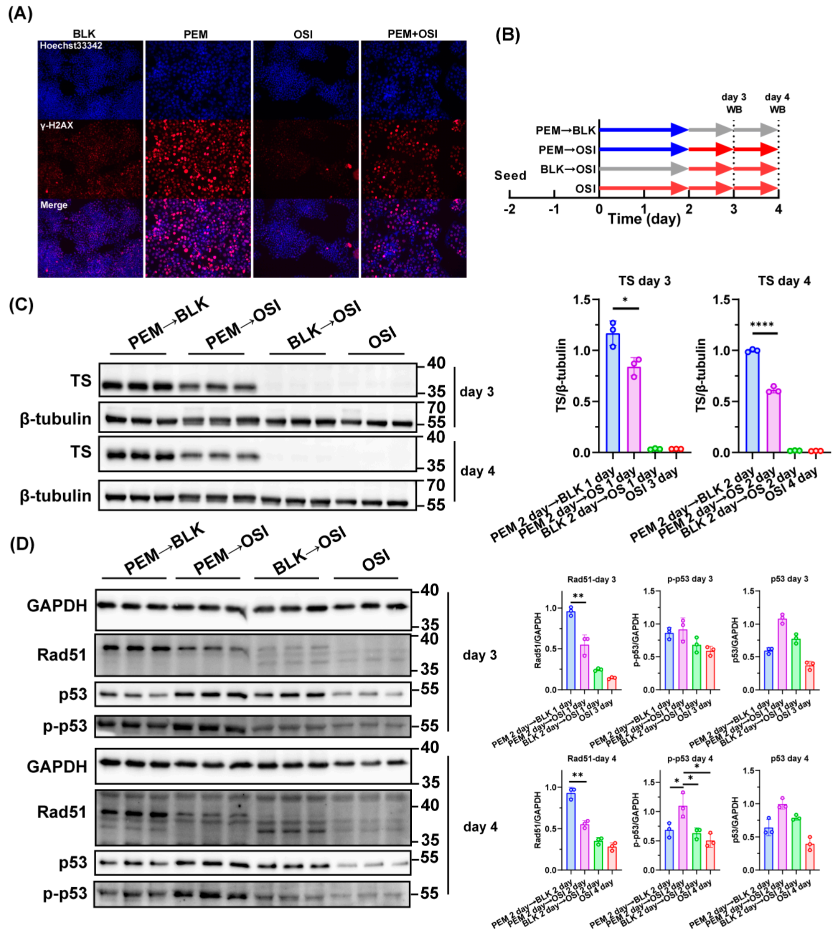

Figure S5 PEM induces DNA damage. Concurrent exposure to OSI attenuates PEM-induced DNA damage. However, OSI downregulates Rad51 and TS, potentially enhancing PEM-induced DNA damage in sequential combination therapy. (A) γ-H2AX immunofluorescence images of HCC827 cells treated for 72 hours with: 1 μM PEM, 20 nM OSI, or PEM+OSI co-treatment. **(B)** Schematic diagram of treatment protocols for panels C and D (n = 3). **(C-D)** Protein expression levels of Rad51, TS, pP53, and total p53 following sequential treatments as outlined in panel B. PEM concentration: 1 μM; OSI concentration: 20 nM (n = 3).

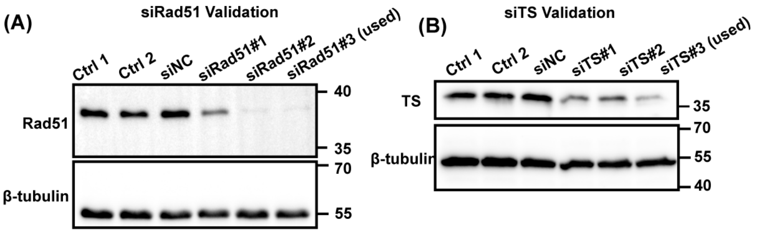

Figure S6 Validation of siRNA knockdown efficiency for Rad51 and TS.

3. Section S3: Corresponding Western Blot image for figure 6, 7, 8, 9

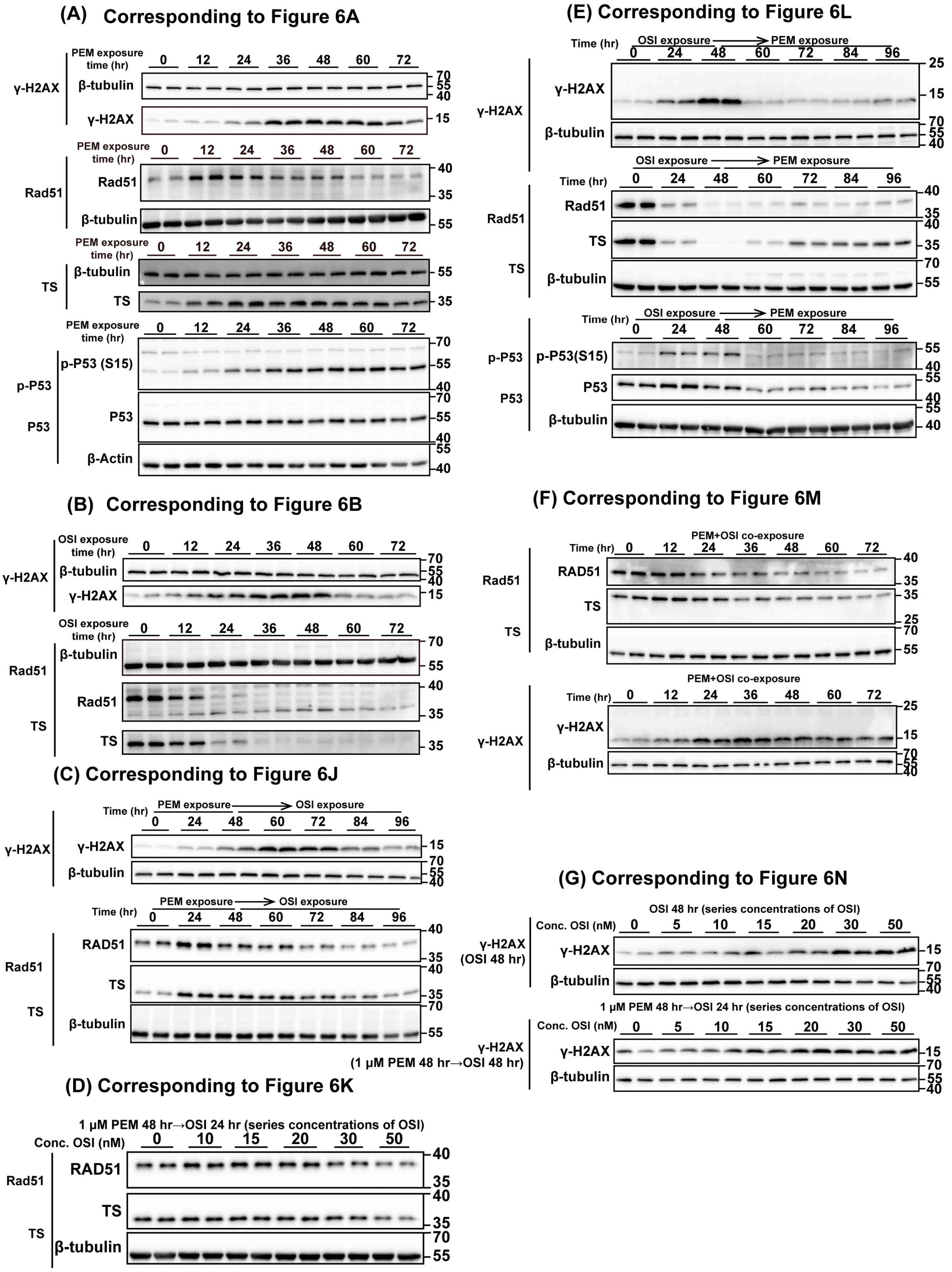

Figure S7: Corresponding WB image for Figure 6.

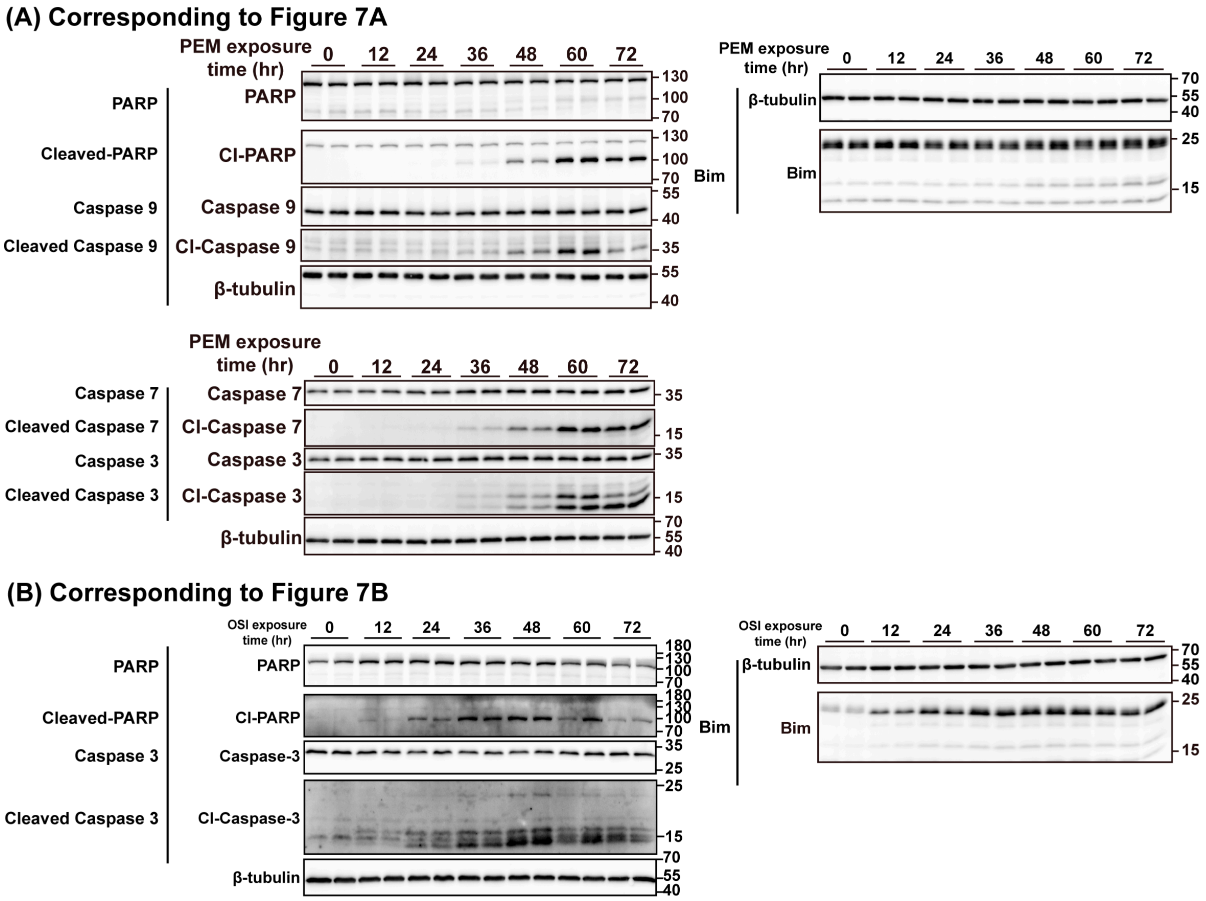

Figure S8: Corresponding WB image for Figure 7.

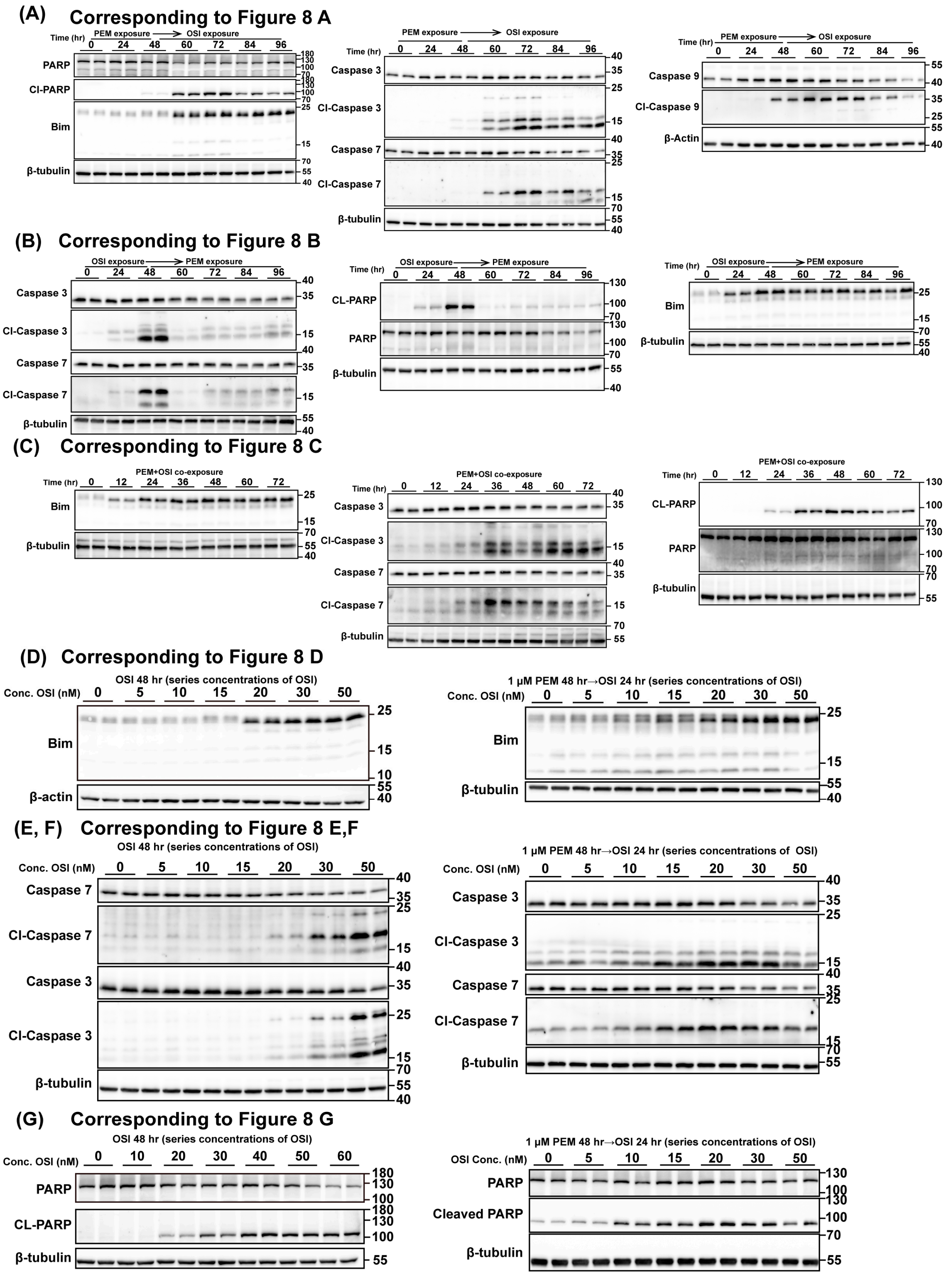

Figure S9: Corresponding WB image for Figure 8.

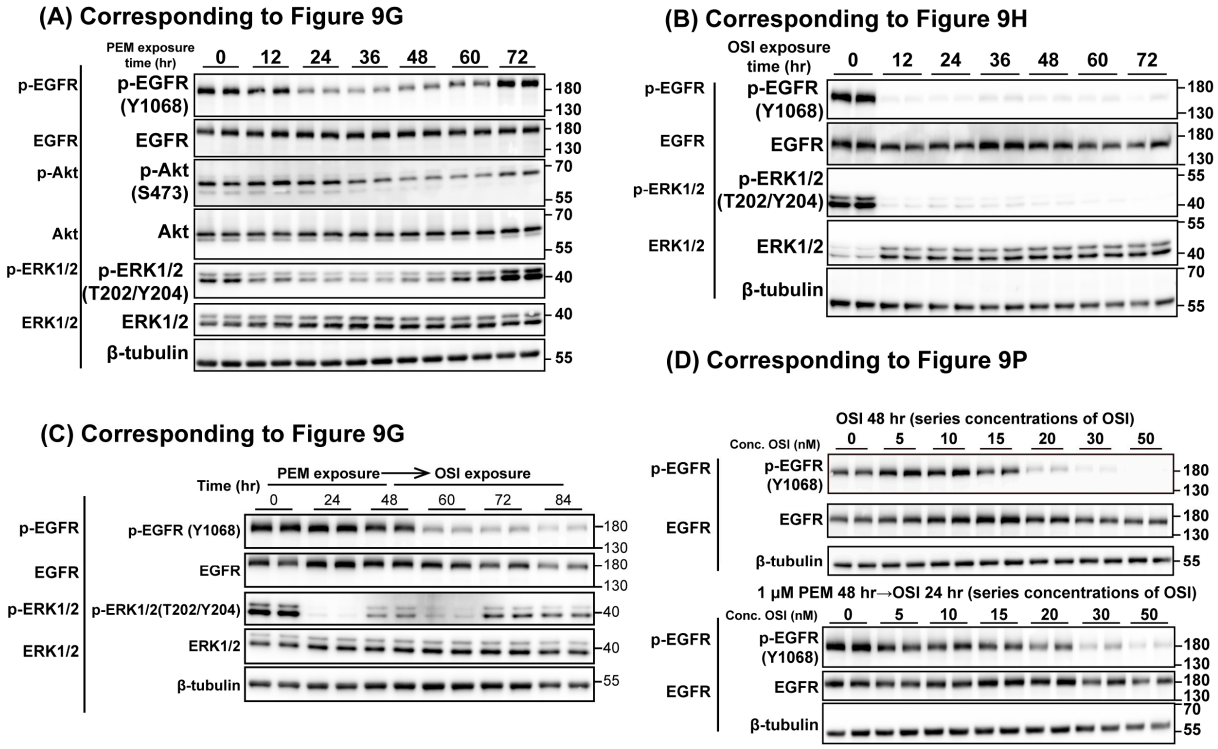

Figure S10: Corresponding WB image for Figure 9.

4. Section S4: Considerations in Concentration Selection for *In Vitro* Assays

The in vitro PEM and OSI concentration used in this study were all within clinically translatable drug exposure levels, and their experimental concentration were selected for the following reasons:

4.1 Consideration in PEM concentration selection

The pharmacokinetic (PK) properties of PEM in NSCLC patients can be referenced from a population pharmacokinetic study of PEM (These literatures indicate that the median plasma concentrations of PEM was initiated at 100 μM and maintained above 0.15 μM for about 48 hr) [7]. PEM was administered intravenously Q21D at a high dose (500 mg/m²) due to its very short half-life (3.5 hours in humans), achieves a plasma concentration of approximately 100 μM immediately after injection. This high dosing strategy is necessary to sustain therapeutic plasma concentrations (e.g., ≥ 0.15 μM) for a period. In NSCLC patients, PEM maintains plasma concentrations above 0.15 μM for up to 48 hours. This pharmacokinetic profile is the rationale for our comparison of 24-hour versus 48-hour effective PEM exposure in the PEM→OSI sequential strategy to assess synergistic efficacy in Figure 1 and Figure 2. The maximum PEM concentrations evaluated in Figure 2 was 0.9 μM, well within the clinically achievable plasma concentration range for PEM.

In our study on the pharmacodynamic interactions between PEM and OSI (i.e. Figures 6, 7, 8, 9), we selected a PEM concentration of 1 μM. This concentration represents the time-averaged mean of the dynamic plasma drug concentration (PEM) observed in actual clinical patients. The specific calculation method is as follows, data were extracted from above mentioned PEM's population pharmacokinetic study [7].

$$\overline{C_{\text{0-168}}} = \frac{AUC_{\text{0-168 h}}}{\tau_{\text{0-168 hr}}} = \frac{[106.3\ (\text{mg·hr/L})]}{168\ \text{hr}} = 0.633\ \text{mg/L} = 633\ \mu\text{g}/L = \frac{633\ \mu\text{g/L}}{427.4\ \text{g/mol}} = 1.48\ \mu\text{M}$$

Where $\overline{C_{\text{0-168}}}$ represent the time averaged plasma concentration for PEM within 0-168 hr., $AUC_{\text{0-168 h}}$ represent the Area Under the Curve (AUC) for PEM plasma-concentration curve cross 0-168 hr for a BSA 1.7 m^2 CrCl: 89.5 ml/min subject, τ refers to the 168-hour time span of the plasma concentration-time curve.

4.2 Consideration in OSI concentration selection

The pharmacokinetic properties of OSI in NSCLC patients can be referenced from a population pharmacokinetic study of OSI combined with PEM-containing chemotherapy (These literatures indicate that plasma concentrations fluctuated between 100 nM and 1000 nM following continuous administration of OSI.) [8-10] regimens, as well as a study on physiologically based pharmacokinetic (PBPK) modeling of OSI (This literature indicates that the observed plasma concentration fluctuated between 200 nM and 1000 nM following continuous administration of OSI.) [11]. OSI, administered as a daily oral medication, maintains plasma drug concentrations through continuous dosing, fluctuating between 100 and 1000 nM. However, OSI has a high plasma protein binding rate (approximately 95%), with a free fraction (unbound fraction) of about 5.35% [12], This means the clinically free (unbound) concentration of OSI fluctuates between 10 and 50 nM, and all OSI concentrations used in this study fall within this range(Figure 2 and Figure 6,7,8,9).

Notably, in clinical practice, the free concentration of OSI undergoes dynamic changes and remains at low levels for a period. In tumor cells pre-treated with PEM for 48 hours, the EC50 for OSI-activated DNA damage and apoptotic signals is significantly reduced (Figure 6N, Figure 8D, E, F, G). This indicates that even when subsequently treated with relatively low free concentrations of OSI, significant DNA damage signals and apoptotic signals can still be induced. This demonstrates that under the PEM→OSI sequential treatment strategy, despite dynamic changes in OSI free concentration, these dynamic concentrations are sufficient to continuously elicit potent DNA damage and apoptotic effects. In contrast, OSI monotherapy may only effectively induce these signals during limited periods of relatively high free concentration.

5. Section S5: STR certification for NSCLC cell lines used in this study

GENETIC TESTING BIOTECHNOLOGY

Cell Line Authentication Service
STR Profile Report

Sales Order: 230510B

Loci	Test Results for Submitted Sample – Query Profile: HCC827			ExPASy Reference Database Profile – Database Profile: HCC827	
Amelogenin	X			X	
D3S1358	17				
D5S818	12			12	
D2S1338	17	24			
TPOX	8			8	
CSF1PO	11			11	
Penta D	14				
TH01	6			6	
vWA	18			18	
D7S820	11	12		11	12
D21S11	31				
Penta E	20				
D10S1248	13	17			
D8S1179	12				
D1S1656	12	17	18		
D18S51	13				
D12S391	17				
D6S1043	11	12			
D19S433	14				
D16S539	12			12	
D13S317	9			9	
FGA	22	24			

The allele match algorithm compares the 8 core loci plus amelogenin only, even though alleles from all loci will be reported when available.
Note: Loci highlighted in grey (8 core STR loci plus Amelogenin) can be made public to verify cell identity. In order to protect the identity of the donor, ***please do not publish*** *the allele calls from all the STR loci tested.*
The sample match is based on the reference data available at the time of comparison.

Explanation of Test Results
Cell lines with ≥80% match are considered to be related; i.e., derived from a common ancestry. Cell lines with between a 55% to 80% match require further profiling for authentication of relatedness.

☐ The submitted sample profile is human, but not a match for any profile in the ExPASy STR database.

☑ The submitted profile is an exact match for the following human cell line(s) in the ExPASy STR database (8 core loci plus Amelogenin): HCC827

☐ The submitted profile is similar to the following ExPASy human cell line(s):

e-Signature Technician: Digitally signed by Xuekun Chen
DN: cn=Xuekun Chen, o=Genetic Testing Biotechnology (Suzhou), ou=DNA Typing Section, email=order@jsdna.org, c=CN
Date: 2023.05.10 15:38:59 +08'00'

e-Signature Reviewer: Digitally signed by Xiankun Zhao
DN: cn=Xiankun Zhao, o=Genetic Testing Biotechnology (Suzhou), ou=Supervision Section, email=service@jsdna.org, c=CN
Date: 2023.05.10 15:39:29 +08'00'

CELL LINE AUTHENTICATION

More information

Addendum: Electropherogram for the customer's sample set 1 of 1

For Research Use ONLY — Page 2 of 3 — Ver. 3.1.2

Figure S11: STR certification for HCC827

GENETIC TESTING BIOTECHNOLOGY

Cell Line Authentication Service
STR Profile Report

Sales Order: 230508A

	Test Results for Submitted Sample		ExPASy Reference Database Profile	
Loci	Query Profile: PC-9		Database Profile: PC-9	
Amelogenin	X		X	
D3S1358	16			
D5S818	11		11	
D2S1338	19	20		
TPOX	11		11	
CSF1PO	11		11	
Penta D	9	13		
TH01	7		7	
vWA	17		17	
D7S820	10	11	10	11
D21S11	29	30		
Penta E	11	15		
D10S1248	13			
D8S1179	11	15		
D1S1656	15	18.3		
D18S51	15			
D12S391	18			
D6S1043	13	19		
D19S433	13	15.2		
D16S539	9		9	
D13S317	8		8	
FGA	23			

The allele match algorithm compares the 8 core loci plus amelogenin only, even though alleles from all loci will be reported when available.
Note: Loci highlighted in grey (8 core STR loci plus Amelogenin) can be made public to verify cell identity. In order to protect the identity of the donor, ***please do not publish*** *the allele calls from all the STR loci tested.*
The sample match is based on the reference data available at the time of comparison.

Explanation of Test Results

Cell lines with ≥80% match are considered to be related; i.e., derived from a common ancestry. Cell lines with between a 55% to 80% match require further profiling for authentication of relatedness.

☐ The submitted sample profile is human, but not a match for any profile in the ExPASy STR database.

☑ The submitted profile is an exact match for the following human cell line(s) in the ExPASy STR database (8 core loci plus Amelogenin): PC-9

☐ The submitted profile is similar to the following ExPASy human cell line(s):

e-Signature Technician: Digitally signed by Xuekun Chen
DN: cn=Xuekun Chen, o=Genetic Testing Biotechnology (Suzhou), ou=DNA Typing Section, email=order@jsdna.org, c=CN
Date: 2023.05.08 15:35:08 +08'00'

e-Signature Reviewer: Digitally signed by Xiankun Zhao
DN: cn=Xiankun Zhao, o=Genetic Testing Biotechnology (Suzhou), ou=Supervision Section, email=service@jsdna.org, c=CN
Date: 2023.05.08 15:35:43 +08'00'

GUARANTEE — CELL LINE AUTHENTICATION

More information

Addendum: Electropherogram for the customer's sample set 1 of 1

For Research Use ONLY — Page 2 of 3 — Ver. 3.1.2

Figure S12: STR certification for PC-9

GENETIC TESTING BIOTECHNOLOGY

Cell Line Authentication Service
STR Profile Report

Sales Order: 210817C

Loci	Test Results for Submitted Sample Query Profile: NCI-H1975		DSMZ Reference Database Profile Database Profile: NCI-H1975	
Amelogenin	X		X	
D3S1358	14	15		
D13S317	10	13	10	13
D7S820	8	11	8	11
D16S539	9	12	9	12
Penta E	12	16		
TPOX	8	11	8	11
TH01	7		7	
D2S1338	17			
CSF1PO	12		12	
Penta D	12	13		
D19S433	15	15.2		
vWA	18		18	
D21S11	28			
D18S51	13			
D6S1043	12			
D8S1179	13	16		
D5S818	11	12	11	12
D12S391	17			
FGA	21	24		

The allele match algorithm compares the 8 core loci plus amelogenin only, even though alleles from all loci will be reported when available.

Note: Loci highlighted in grey (8 core STR loci plus Amelogenin) can be made public to verify cell identity. In order to protect the identity of the donor, ***please do not publish*** *the allele calls from all the STR loci tested. The sample match is based on the reference data available at the time of comparison.*

Explanation of Test Results

Cell lines with ≥80% match are considered to be related; i.e., derived from a common ancestry. Cell lines with between a 55% to 80% match require further profiling for authentication of relatedness.

- ☐ The submitted sample profile is human, but not a match for any profile in the DSMZ STR database.
- ☑ The submitted profile is an exact match for the following human cell line(s) in the DSMZ STR database (8 core loci plus Amelogenin): NCI-H1975
- ☐ The submitted profile is similar to the following DSMZ human cell line(s):

e-Signature, Technician: Digitally signed by Xuekun Chen
DN: cn=Xuekun Chen, o=Genetic Testing Biotechnology (Suzhou), ou=DNA Typing Section, email=order@jsdna.org, c=CN
Date: 2021.08.17 16:20:37 +08'00'

GUARANTEE CELL LINE AUTHENTICATION

e-Signature, Reviewer: Digitally signed by Xiankun Zhao
DN: cn=Xiankun Zhao, o=Genetic Testing Biotechnology (Suzhou), ou=Supervision Section, email=service@jsdna.org, c=CN
Date: 2021.08.17 16:21:11 +08'00'

Addendum: Electropherogram for the customer's sample set 1 of 1

For Research Use ONLY   Page 2 of 3   Ver. 3.1.2

Figure S13: STR certification for NCI-H1975

References for the supplementary materials:

1. Tanaka, Kosuke, Helena A. Yu, Shaoyuan Yang, Song Han, S. Duygu Selcuklu, Kwanghee Kim, Shriram Ramani, Yogesh Tengarai Ganesan, Allison Moyer, Sonali Sinha, Yuchen Xie, Kota Ishizawa, Hatice U. Osmanbeyoglu, Yang Lyu, Nitin Roper, Udayan Guha, Charles M. Rudin, Mark G. Kris, James J. Hsieh, and Emily H. Cheng. "Targeting Aurora B Kinase Prevents and Overcomes Resistance to Egfr Inhibitors in Lung Cancer by Enhancing Bim- and Puma-Mediated Apoptosis." *Cancer Cell* 39, no. 9 (2021): 1245-61.e6.
2. Ayestaran, Iñigo, Ana Galhoz, Elmar Spiegel, Ben Sidders, Jonathan R. Dry, Frank Dondelinger, Andreas Bender, Ultan McDermott, Francesco Iorio, and Michael P. Menden. "Identification of Intrinsic Drug Resistance and Its Biomarkers in High-Throughput Pharmacogenomic and Crispr Screens." *Patterns* 1, no. 5 (2020).
3. Yang, Chi-Hwa, Hsiao-Chin Chou, Yu-Ning Fu, Chi-Ling Yeh, Hui-Wen Cheng, Il-Chi Chang, Ko-Jiunn Liu, Gee-Chen Chang, Ting-Fen Tsai, Shih-Feng Tsai, Hui-Ping Liu, Yi-Cheng Wu, Ya-Ting Chen, Shiu-Feng Huang, and Yi-Rong Chen. "Egfr over-Expression in Non-Small Cell Lung Cancers Harboring Egfr Mutations Is Associated with Marked Down-Regulation of Cd82." *Biochimica et Biophysica Acta (BBA) - Molecular Basis of Disease* 1852, no. 7 (2015): 1540-49.
4. Fregni, Matilde, Yari Ciribilli, and Joanna E. Zawacka-Pankau. "The Therapeutic Potential of the Restoration of the P53 Protein Family Members in the Egfr-Mutated Lung Cancer." *International Journal of Molecular Sciences* 23, no. 13 (2022).
5. Fregni, M., Y. Ciribilli, and J. E. Zawacka-Pankau. "The Therapeutic Potential of the Restoration of the P53 Protein Family Members in the -Mutated Lung Cancer." *International Journal of Molecular Sciences* 23, no. 13 (2022).
6. Zhou, Yaodong, Dongdong Guo, and Yixin Zhang. "Association of Microrna-21 with P53 at Mutant Sites R175h and R248q, Clinicopathological Features, and Prognosis of Nsclc." *Molecular Therapy - Oncolytics* 19 (2020): 208-17.
7. Cao, Peng, Wei Guo, Jun Wang, Sanlan Wu, Yifei Huang, Yang Wang, Yani Liu, and Yu Zhang. "Population Pharmacokinetic Study of Pemetrexed in Chinese Primary Advanced Non-Small Cell Lung Carcinoma Patients." *Frontiers in Pharmacology* 13 (2022).
8. Yang, Jincheng, Damilola Olabode, Aarti Sawant‐Basak, Richard Baldry, Karthick Vishwanathan, Srinivas Bachina, Alexandar Todd, Dana Ghiorghiu, Yuri Rukazenkov, Diansong Zhou, and Azar Shahraz. "Population Pharmacokinetics and Exposure‐Response Analysis of First‐Line Osimertinib Plus Chemotherapy in Patients with Egfr‐Mutated Advanced Nsclc." *Clinical Pharmacology & Therapeutics* (2025).
9. Brown, Kathryn, Craig Comisar, Han Witjes, John Maringwa, Rik de Greef, Karthick Vishwanathan, Mireille Cantarini, and Eugène Cox. "Population Pharmacokinetics and Exposure‐Response of Osimertinib in Patients with Non‐Small Cell Lung Cancer." *British Journal of Clinical Pharmacology* 83, no. 6 (2017): 1216-26.
10. Johnson, Martin, Yu‐Wei Lin, Henning Schmidt, Mikael Sunnaker, Eline Van Maanen, Xiangning Huang, Yuri Rukazenkov, Helen Tomkinson, and Karthick Vishwanathan. "Population Pharmacokinetics of Osimertinib in Patients with Non‐Small Cell Lung Cancer." *Pharmacology Research & Perspectives* 13, no. 3 (2025).
11. Pilla Reddy, Venkatesh, Michael Walker, Pradeep Sharma, Peter Ballard, and Karthick

Vishwanathan. "Development, Verification, and Prediction of Osimertinib Drug–Drug Interactions Using Pbpk Modeling Approach to Inform Drug Label." *CPT: Pharmacometrics & Systems Pharmacology* 7, no. 5 (2018): 321-30.

12. Colclough, Nicola, Kan Chen, Peter Johnström, Nicole Strittmatter, Yumei Yan, Gail L. Wrigley, Magnus Schou, Richard Goodwin, Katarina Varnäs, Sally J. Adua, Minghui Zhao, Don X. Nguyen, Gareth Maglennon, Peter Barton, James Atkinson, Lin Zhang, Annika Janefeldt, Joanne Wilson, Aaron Smith, Akihiro Takano, Ryosuke Arakawa, Mikhail Kondrashov, Jonas Malmquist, Evgeny Revunov, Ana Vazquez-Romero, Mohammad Mahdi Moein, Albert D. Windhorst, Natasha A. Karp, M. Raymond V. Finlay, Richard A. Ward, James W. T. Yates, Paul D. Smith, Lars Farde, Zack Cheng, and Darren A. E. Cross. "Preclinical Comparison of the Blood–Brain Barrier Permeability of Osimertinib with Other Egfr Tkis." *Clinical Cancer Research* 27, no. 1 (2021): 189-201.
